# An organoid-based CRISPR-Cas9 screen for regulators of intestinal epithelial maturation and cell fate

**DOI:** 10.1126/sciadv.adg4055

**Published:** 2023-07-12

**Authors:** Stine L. Hansen, Hjalte L. Larsen, Laura M. Pikkupeura, Grzegorz Maciag, Jordi Guiu, Iris Müller, Ditte L. Clement, Christina Mueller, Jens Vilstrup Johansen, Kristian Helin, Mads Lerdrup, Kim B. Jensen

**Affiliations:** ^1^ Novo Nordisk Foundation Center for Stem Cell Medicine, reNEW, Faculty of Health and Medical Sciences, University of Copenhagen, DK-2200 Copenhagen N, Denmark.; ^2^ Biotech Research and Innovation Centre, University of Copenhagen, Ole Maaloes Vej 5, DK-2200 Copenhagen N, Denmark.; ^3^ Institut d’Investigació Biomèdica de Bellvitge–IDIBELL, 3a planta, Av. Granvia de l’Hospitalet 199, 08908 Hospitalet de Llobregat, Spain.; ^4^ The DNRF Center for Chromosome Stability, Department of Cellular and Molecular Medicine, University of Copenhagen, DK-2200 Copenhagen N, Denmark.

## Abstract

Generation of functionally mature organs requires exquisite control of transcriptional programs governing cell state transitions during development. Despite advances in understanding the behavior of adult intestinal stem cells and their progeny, the transcriptional regulators that control the emergence of the mature intestinal phenotype remain largely unknown. Using mouse fetal and adult small intestinal organoids, we uncover transcriptional differences between the fetal and adult state and identify rare adult-like cells present in fetal organoids. This suggests that fetal organoids have an inherent potential to mature, which is locked by a regulatory program. By implementing a CRISPR-Cas9 screen targeting transcriptional regulators expressed in fetal organoids, we establish *Smarca4* and *Smarcc1* as important factors safeguarding the immature progenitor state. Our approach demonstrates the utility of organoid models in the identification of factors regulating cell fate and state transitions during tissue maturation and reveals that SMARCA4 and SMARCC1 prevent precocious differentiation during intestinal development.

## Teaser

Functional perturbation screen in intestinal organoids uncovers that SWI/SNF components safeguard tissue maturation.

## INTRODUCTION

During organ development, timely regulation of tissue maturation is essential for balancing growth with the emergence of differentiated cell types and the acquisition of tissue functionality. The developing intestinal tube initially transitions from a pseudo-stratified epithelium into a simple epithelium consisting of rudimentary villi and intervillous domains (*1*). The intervillous domains subsequently give rise to epithelial invaginations that will later form the adult crypts, the niche of adult intestinal stem cells. These morphological rearrangements govern the emergence of adult intestinal stem cells, as equipotent fetal progenitors are subjected to instructive cues from the emerging stem cell niche (*2*). While numerous studies have addressed the morphological events during intestinal development (*3*) and the regulation of adult intestinal stem cell behavior (*4*), little is known about the transcriptional regulation associated with the transition from a fetal to an adult cell state. In vivo studies have demonstrated that the master regulator of hindgut identity CDX2 navigates open chromatin to enable expression of HNF4A/Y, which subsequently drives the maturation of the intestinal epithelium (*5*–*7*). The inductive capacity of CDX2 and HNF4A in the specification of the adult intestinal epithelium was further supported by the direct reprogramming of embryonic fibroblasts toward an adult intestinal epithelium by ectopic expression of CDX2, HNF4A, GATA6, and FOXA3 (*8*). In addition, the histone demethylase LSD1 was recently demonstrated to be required for Paneth cell specification and maturation of the intestinal epithelium, as its deletion enforced the acquisition of a fetal-like state in the adult intestinal epithelium (*9*). In contrast to the above factors driving intestinal maturation, two complementary studies have demonstrated a role for the transcription factor BLIMP1/PRDM1 in safeguarding the fetal state, as in vivo deletions led to precocious tissue maturation (*10*, *11*). However, beyond the above, there is scarce knowledge regarding the transcriptional and epigenetic factors controlling cell fate in the developing intestinal epithelium.

The organoid technology has been instrumental in studies of the intestinal epithelium by facilitating the culture of primary epithelial cells. Here, both the adult and fetal intestinal epithelium can be stably maintained under defined in vitro conditions that maintain the cell type composition and maturation status of the parental tissue (*12*–*15*). While adult intestinal organoids exhibit a budding phenotype, fetal intestinal progenitors grow as symmetrical fetal enterospheres (FEnS). Once established, FEnS are stable in long-term culture and do not spontaneously mature to their adult counterparts (*14*). Given that adult organoids (aOrgs) and FEnS can be stably maintained under identical in vitro conditions, we reasoned that this could serve as a tractable system for interrogating factors safeguarding the fetal state or promoting epithelial maturation. Using the CRISPR-Cas9 technology and a custom-designed small guide RNA (sgRNA) library targeting functional domains in transcriptional regulators specifically up-regulated in FEnS, we uncover factors required for either repressing or facilitating intestinal maturation and demonstrate a role for the switch/sucrose nonfermentable (SWI/SNF) complex components SMARC4 and SMARCC1 in the process of tissue maturation. To our knowledge, this represents the first reported application of a CRISPR-Cas9 screen in organoids to identify regulators of tissue maturation, hence demonstrating a novel application of the organoid technology.

## RESULTS

### FEnS have intrinsic maturation potential

**Fig. 1.**
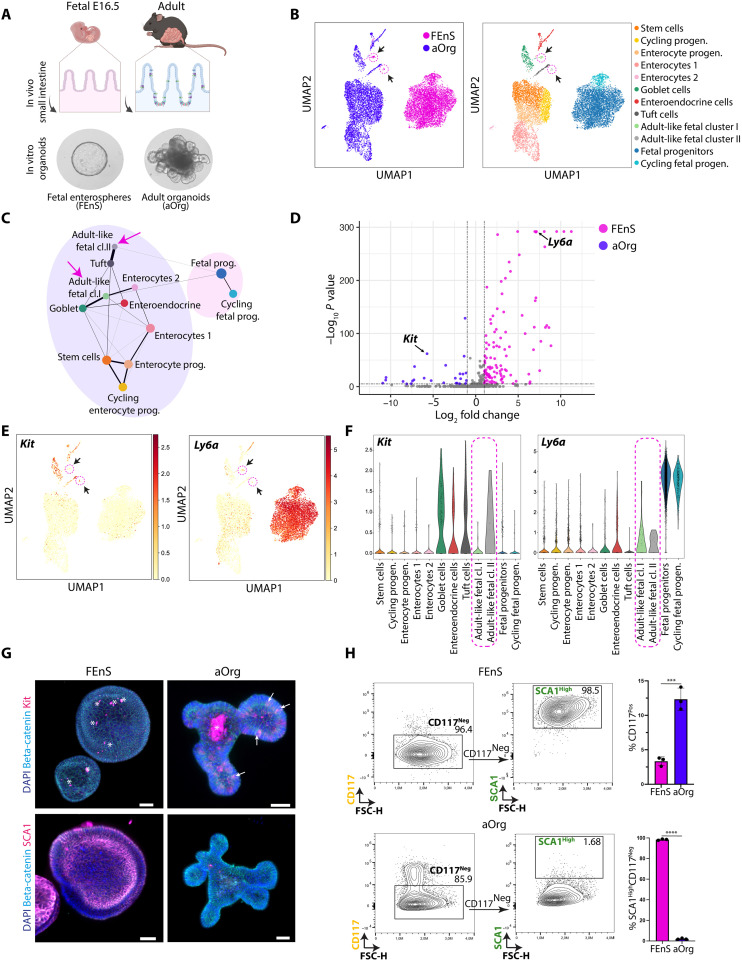
Characterization of cellular heterogeneity and marker expression in intestinal organoids. (**A**) Illustration of mouse intestinal organoid phenotypes derived from distinct stages. Epithelial cells isolated from the fetus at E16.5 give rise to FEnS in three-dimensional (3D) culture, while cells isolated from the adult intestine give rise to budding aOrgs. (**B**) Uniform Manifold Approximation and Projection (UMAP) plot of cells from FEnS or aOrgs following scRNA-seq. Left: FEnS (pink) and aOrg (blue). Right: Annotation of cell types based on canonical marker expression (see fig. S1A). Arrows indicate adult-like clusters of cells derived from FEnS. (**C**) PAGA trajectory of clusters from (B). The size of the nodes is proportional to the number of cells in each cluster, and the thickness of the edges represents confidence in transcriptional connection. Arrows indicate adult-like fetal clusters I and II. (**D**) Volcano plot of DE surface marker genes between the fetal progenitor cluster (pink) and adult secretory clusters (blue). *x* axis: Log_2_ fold change (LFC); *y* axis: Log_10_ *P* value. *Kit* (adult marker) and *Ly6a* (fetal marker) are highlighted. Outliers with 15 < LFC < −15 and high *P* values were removed. (**E**) UMAP plot showing expression levels of *Kit* (left) and *Ly6a* (right). Arrows indicate the adult-like fetal clusters I and II. (**F**) Violin plots of *Ly6a* and *Kit* expression across cell type clusters of fetal and adult cells. (**G**) Immunofluorescence images of CD117 expression (top, scale bars, 100 μm) and SCA1 expression (bottom, scale bars, 50 μm) in 3D FEnS and aOrgs. Arrows = CD117^+^ cells; asterisks = debris. (**H**) Flow cytometry–based contour plots of CD117 (*Kit*) and SCA1 (*Ly6a*) expression in FEnS and aOrgs. Quantification of indicated populations is summarized in the bar plot to the right (*n* = 3). Error bars indicate SD, unpaired Student’s *t* test. ***P < 0.001 and ****P < 0.0001. DAPI, 4′,6-diamidino-2-phenylindole; FSC, forward scatter.

To characterize the cellular composition of organoid cultures derived from the fetal and adult small intestinal epithelium at single-cell resolution, we performed single-cell RNA sequencing (scRNA-seq) on established cultures of FEnS and aOrg from the proximal part of the small intestine (Fig. 1A). As expected, all major cell types present in the intestinal epithelium in vivo could be identified in aOrgs. In line with the observation that the parental embryonic day (E) 16.5 intestinal epithelium consists of immature equipotent progenitor cells (*2*), FEnS largely consisted of a homogenous population of undifferentiated progenitor cells (Fig. 1B). We identified two cell populations in FEnS with distinct transcriptional profiles from the progenitor cells, clustering with the adult secretory lineages. These cells had notable transcriptional similarities with goblet and tuft cells in the adult epithelium, respectively, and were termed adult-like fetal clusters I and II (Fig. 1, B and C). aOrg formation is characterized by distinct phases of growth, where the initial phase is associated with the expression of fetal-like markers and a spherical appearance. The transition into a budding organoid structure is driven by the first symmetry-breaking event characterized by the emergence of a secretory cell (*16*). The presence of a small population of adult-like secretory cells in FEnS and the absence of spontaneous conversion of the culture toward aOrgs suggested that transcriptional or epigenetic barriers prevented the complete transition towards a mature adult state. We therefore reasoned that a functional CRISPR-Cas9 screen could identify transcriptional regulators that constitute a barrier for the transition from the fetal to the adult state.

### Identification of markers discriminating the fetal and adult cell states

To identify cell surface markers discriminating FEnS and aOrgs as a functional readout of their maturation status, we took advantage of the scRNA-seq dataset. Analysis of differentially expressed genes (DEGs) between the fetal progenitor cluster and the secretory clusters derived from aOrgs allowed us to identify 1960 DEGs [log_2_ fold change (LFC) > 1; *P* < 0.05]. Filtering the list of DEGs for cell surface–associated genes allowed us to identify *Ly6a* (SCA1) and *Kit* (cKIT/CD117) as markers of fetal progenitors and secretory cells, respectively (Fig. 1, D to F). SCA1 has previously been described as a marker of the fetal intestinal state and as a marker of adult intestinal cells undergoing transient fetal-like reprogramming during tissue injury (*17*). Similarly, CD117 is a well-known marker of secretory cells in the adult small intestine and colon (*18*). Analysis of expression of the two markers further supported these observations and demonstrated specific SCA1 expression in FEnS (Fig. 1, G and H), and detection of CD117 in a subset of cells in aOrgs, and in rare cells in FEnS (Fig. 1, G and H). A combination of CD117 and SCA1 thus offers high discrimination power between aOrgs and FEnS that aligns with their maturation status.

### Implementation of a CRISPR-Cas9 screen for regulators of intestinal epithelial maturation

**Fig. 2.**
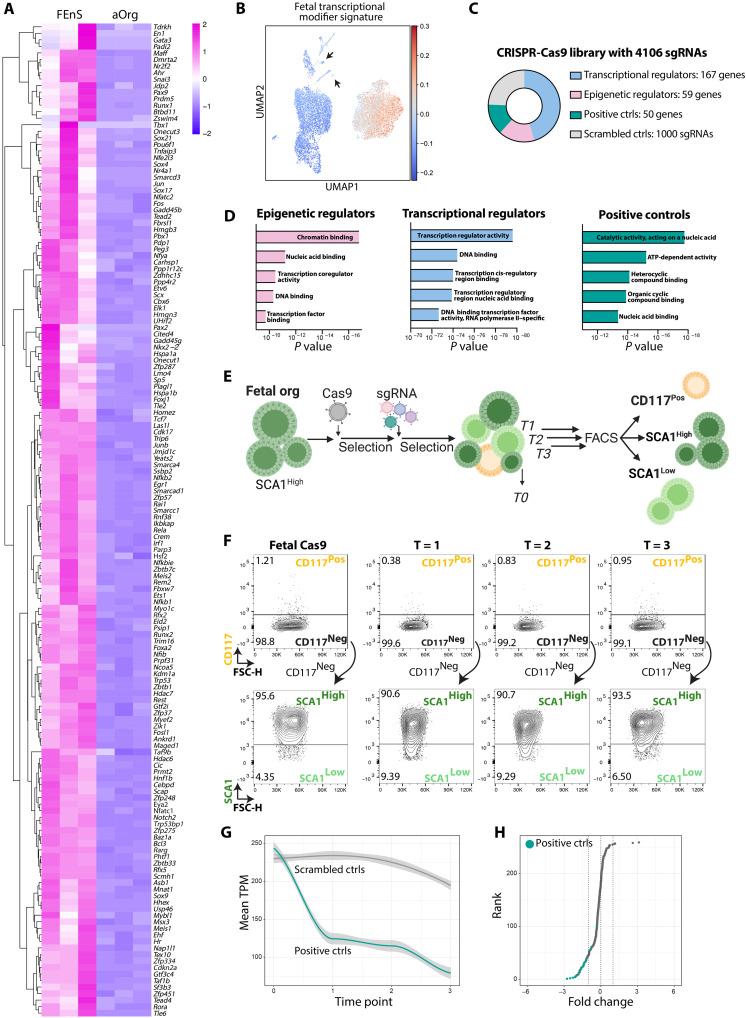
Implementation of a targeted CRISPR-Cas9 screen in FEnS. (**A**) Heatmap of transcription factors and epigenetic regulators differentially expressed between FEnS and aOrgs (*n* = 3, log_2_ FC > 1, FDR < 0.05) (see fig. S2A). (**B**) UMAP plot showing relative enrichment of fetal transcriptional modifiers in FEnS and aOrgs (see Fig.S2B). Arrows indicate adult-like fetal clusters I and II. (**C**) Composition of the targeted CRISPR-Cas9 sgRNA library. (**D**) Enriched biological process Gene Ontology terms from indicated categories of the genes included in the sgRNA library: epigenetic regulators, transcriptional regulators, and positive controls. *x* axis: *P* value. (**E**) Illustration of the experimental flow of the CRISPR-Cas9 screen in FEnS. (**F**) Flow cytometry contour plots showing CD117 and SCA1 expression in parental FEnS expressing Cas9, and the expression at the three analytical time points of the screen. Three populations were sorted at each time point: CD117^Pos^, CD117^Neg^SCA1^High^, and CD117^Neg^SCA1^Low^. (**G**) Normalized transcripts per million (TPM) of scrambled control nontargeting sgRNAs (gray) and positive controls targeting essential genes (teal) during the three time points of the screen. (**H**) Rank score plot of control sgRNAs based on their FC enrichment in T = 1 versus T = 0. Positive controls targeting essential genes are shown in teal. ATP, adenosine 5´-triphosphate.

Given that FEnS can be stably maintained in culture in their immature state without spontaneous conversion to aOrgs, we hypothesized that FEnS expressed transcriptional regulators safeguarding their fetal identity. To generate a list of candidate transcriptional and epigenetic regulators that could potentially safeguard the fetal state, we reanalyzed a population-based expression analysis dataset of FEnS and aOrg (*19*). The analysis revealed that 335 transcription factors and epigenetic regulators were differentially expressed between FEnS and aOrgs, of which 167 transcription factors and 59 epigenetic regulators were up-regulated in the fetal state [LFC > 1, false discovery rate (FDR) < 0.05] (Fig. 2A and fig. S2A). To validate the expression of these factors in the various cell types in FEnS and aOrgs, we compiled a fetal transcriptional regulator signature and integrated this with our scRNA-seq dataset, revealing higher expression of this signature in FEnS (Fig. 2B). The two adult-like clusters arising from FEnS expressed very low levels of the identified transcriptional regulator signature otherwise enriched in FEnS (Fig. 2B and fig. S2B). This further highlights the adult-like phenotype associated with these clusters.

We hypothesized that, within this pool of transcriptional regulators, some would safeguard the fetal identity. If this was the case, mutating these fetal transcriptional regulators could be sufficient to interfere with cell state changes and thereby influence the organoid composition and potential maturation. To address this, we designed a custom sgRNA library targeting the 226 transcriptional regulators up-regulated in FEnS with 10 to 12 sgRNAs covering regions encoding functional protein domains (*20*, *21*). The library also contained sgRNAs targeting 50 genes essential for basic cellular functions as positive controls and 1000 scrambled sgRNAs as negative controls (Fig. 2C). Gene Ontology term analyses confirmed that the identified transcription factors included in the sgRNA library were involved in biological processes such as transcriptional regulator activity and DNA binding, while the epigenetic factors were enriched in chromatin binding and nucleic acid binding processes (Fig. 2D).

To perform the CRISPR-Cas9–based screen, we expressed constitutive active CAS9 in FEnS and transduced these with the sgRNA library at a multiplicity of infection (MOI) = 0.3 and a coverage of >400× per sgRNA. A baseline reference sample for the pool of sgRNAs was collected at time point T = 0. The transduced cells were subsequently passaged three times and, at each passage, ^2^/_3_ of the cultured cells were sorted to isolate SCA11^High^CD117^Neg^ fetal progenitors, SCA1^Low^CD117^Pos^ secretory cells, and SCA1^Low^CD117^Neg^ cells putatively transitioning from the fetal state toward an adult-like state (Fig. 2E). At each time point, we identified SCA1^Low^CD117^Pos^ secretory cells along with SCA1^Low^CD117^Neg^ cells transitioning cells (Fig. 2F). The DNA from each of the isolated cell populations was subjected to targeted sequencing to quantify the abundance of individual sgRNAs across the three time points relative to the reference T = 0 sample. As expected, sgRNAs targeting the essential genes associated with primary cell functions were rapidly depleted already at T = 1 (Fig. 2G), whereas the scrambled control sgRNAs were maintained stably over the three passages (Fig. 2G).

### Fetal cells with mutations in Smarca4 and Smarcc1 gain adult-like characteristics

To perform a robust and stringent analysis of the effect of the CRISPR-Cas9–mediated disruption of individual genes, we harnessed the power of our multi-sgRNA per gene approach to quantifying the combined effect of individual sgRNAs targeting the same gene. Here, we computed a single rank score for each gene based on aggregated ranks of FCs and *P* values in the different parameters examined during the screen. This again revealed that cells with disrupted positive control genes were rapidly depleted at the first time point of analysis compared to the T = 0 reference, while scrambled control guides were neutrally maintained across the course of the screen (Fig. 2H).

**Fig. 3.**
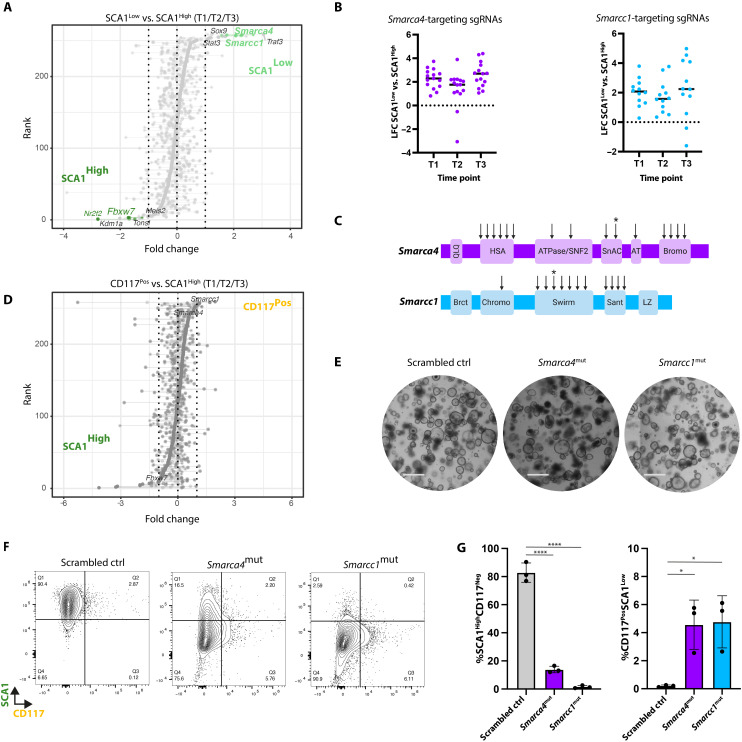
Mutations in *Smarca4* and *Smarcc1* induce transition toward an adult-like state. (**A**) Rank score plot of genes targeted by individual sgRNAs based on their FC in the SCA1^Low^CD117^Neg^ versus SCA1^High^CD117^Neg^ gate for all three time points versus T = 0. *x* axis: FC; *y* axis: rank score according to the combined effect of all sgRNAs targeting the indicated genes. Gene names indicated the top 5 ranked genes in the SCA1^High^ (dark green) and SCA1^Low^ (light green) populations. (**B**) Dot plot of the LFC of sgRNAs found in SCA1^Low^CD117^Neg^ versus SCA1^High^CD117^Neg^ gates targeting *Smarca4* (left) and *Smarcc1* (right) across the three time points. *x* axis: time point; *y* axis: LFC of the normalized sgRNA abundance in SCA1^Low^CD117^Neg^ versus SCA1^High^CD117^Neg^ gates. (**C**) Schematics of the functional domains targeted by sgRNAs (arrows) for *Smarca4* (top) and *Smarcc1* (bottom). Asterisks indicate sgRNAs used for the generation of mutant lines for validation experiments. (**D**) Rank score plot of genes targeted by individual sgRNAs based on their FC in the CD117^Pos^ versus SCA1^High^CD117^Neg^ gate for all three time points versus T = 0. *x* axis: FC, *y* axis: rank score according to the combined effect of all sgRNAs targeting the indicated genes. Genes of interest are indicated by name. (**E**) Bright-field images of FEnS representing the scrambled control line and lines carrying mutations in *Smarca4* and *Smarcc1*. Scale bars, 1 mm. (**F**) Flow cytometry contour plots showing CD117 and SCA1 expression in scrambled control, *Smarca4*^mut^, and *Smarcc1*^mut^ fetal organoids. (**G**) Bar plot of percentage of SCA1^High^CD117^Neg^ (left) and SCA1^Low^CD117^Pos^ (right) cells in scrambled control, *Smarca4*^mut^, and *Smarcc1*^mut^ FEnS. *n* = 3, error bars indicate SD, unpaired Student’s *t* test. *P < 0.05 and ****P < 0.0001.

To identify genes implicated in the transition from a fetal to an adult state, we compared the sgRNA abundance between the SCA1^Low^CD117^Neg^ and SCA1^High^CD117^Neg^ populations. Here, rank score analysis identified sgRNAs targeting *Smarca4* and *Smarcc1* as top candidates enriched in the SCA1^Low^CD117^Neg^ population (Fig. 3A). Analysis of individual sgRNAs targeting *Smarca4* or *Smarcc1* demonstrated that the majority of sgRNAs, irrespective of the targeted functional domain, displayed a similar enrichment across the three time points (Fig. 3, B and C). Similarly, *Smarca4* and *Smarcc1* were among the top-ranking genes when comparing sgRNAs enriched in the SCA1^Low^CD117^Pos^ gate versus the SCA1^High^CD117^Neg^ populations (Fig. 3D). Thus, using two different parameters discriminating adult and fetal cells, namely, the loss of SCA1 expression and the increased capacity to give rise to CD117^Pos^ cells, fetal progenitors with disruptions in *Smarca4* or *Smarcc1* lost their fetal characteristics and gained an adult-like phenotype. This suggests that SMARCA4 and SMARCC1 play a role in safeguarding the fetal progenitor state by functioning as epigenetic barriers toward maturation.

*Smarca4* (*Brg1*) and *Smarcc1* (*Baf155*) are both components of the SWI/SNF complex which control nucleosome positioning and regulation of the chromatin landscape (*22*). SMARCA4 is one of two catalytic adenosine triphosphatase subunits incorporated into the SWI/SNF complex, whereas SMARCC1 has been reported to have a scaffolding function in the assembly of the many different variants of the multimeric SWI/SNF complex (*22*). Genetic studies have demonstrated that epithelial loss of *Smarca4* during intestinal development leads to precocious epithelial maturation as manifested by accelerated secretory differentiation (*23*). Similarly, the deletion of *Smarca4* in adult intestinal stem cells has been demonstrated to drive stem cell exhaustion (*24*). To validate and explore the role of *Smarca4* and *Smarcc1* in controlling cell state transitions in the organoid models, we generated independent *Smarca4* and *Smarcc1* sgRNA-targeted lines (Fig. 3E and fig.S3, A to D). While the overall morphology did not notably differ between the independent *Smarca4*- and *Smarcc1*-mutated lines (*Smarca4*^mut^ and *Smarcc1*^mut^ hereafter) and control lines transduced with a single-scrambled sgRNA control (Fig. 3E), the mutated lines displayed a significant decrease in the fraction of SCA1-expressing cells and an increased number of CD117^Pos^ cells (Fig. 3, F and G). Hence, mutating these two different components of the SWI/SNF complex confirmed the results from the phenotypic screen.

### Smarca4^mut^ and Smarcc1^mut^ fetal cells adopt an adult small intestinal-like phenotype upon transplantation

**Fig. 4.**
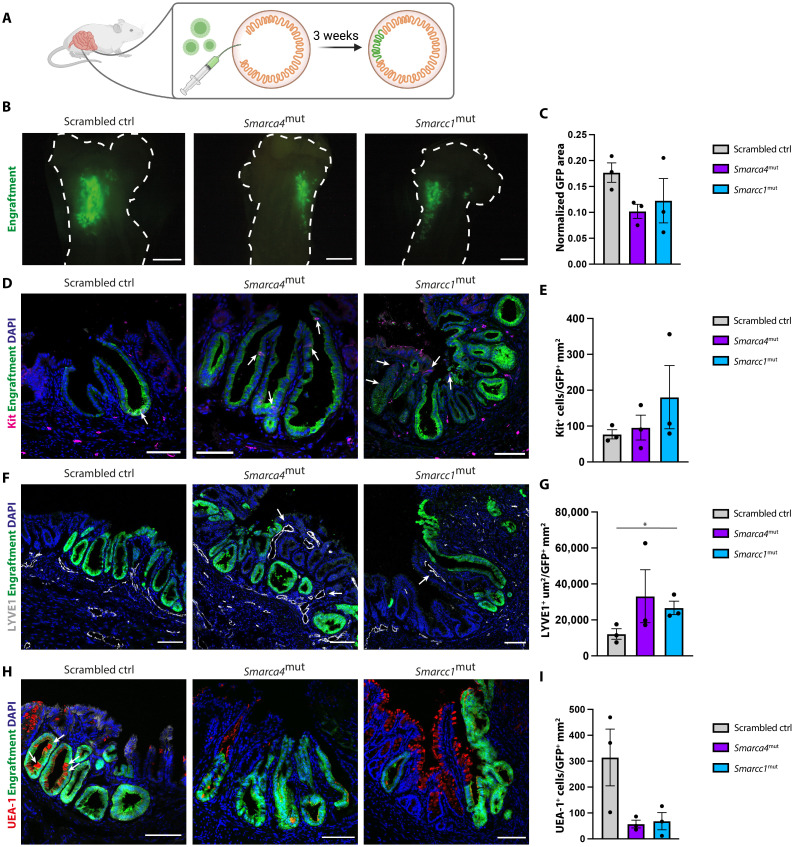
*Smarca4*^mut^ and *Smarcc1*^mut^ fetal organoids obtain an adult-like small intestinal phenotype upon colonic engraftment. (**A**) Schematic of transplantation assay. (**B**) Epi-fluorescence images of representative engrafted areas (GFP, green) in scrambled control, *Smarca4*^mut^, and *Smarcc1*^mut^ (*n* = 3 per organoid line). The dotted line indicates the colonic outline. Scale bars, 4 mm. (**C**) Quantification of engrafted GFP^+^ area in scrambled control, *Smarca4*^mut^, and *Smarcc1*^mut^ normalized to scrambled control engraftment. *n* = 3, error bars indicate SEM. No statistical significance in differences between groups. (**D**) Immunofluorescence staining of Kit (CD117) (red) and DAPI (blue) in engrafted GFP^+^ (green) tissue of scrambled control, *Smarca4*^mut^, and *Smarcc1*^mut^. Scale bars, 100 μm. (**E**) Quantification of CD117^+^ (Kit^+^) cells per GFP^+^ area in scrambled control, *Smarca4*^mut^, and *Smarcc1*^mut^ engrafted tissue. *n* = 3 and error bars indicate SEM. No statistical significance in differences between groups. (**F**) Immunofluorescence staining of LYVE1 (gray) and DAPI (blue) in engrafted GFP^+^ (green) tissue of scrambled control, *Smarca4*^mut^, and *Smarcc1*^mut^. Scale bars, 100 μm. (**G**) Quantification of LYVE1^+^ area per GFP^+^ area in scrambled control, *Smarca4*^mut^, and *Smarcc1*^mut^ engrafted tissue. *n* = 3 and error bars indicate SEM. *P < 0.05, Student’s *t* test. (**H**) Immunofluorescence staining of *Ulex Europaeus* agglutinin 1 (UEA-1) lectin (red) and DAPI (blue) in engrafted GFP^+^ (green) tissue from scrambled control, *Smarca4*^mut^, and *Smarcc1*^mut^ lines. Scale bars, 100 μm. (**I**) Quantification of UEA-1^+^ cells per GFP area in scrambled control, *Smarca4*^mut^, and *Smarcc1*^mut^ engrafted tissue. *n* = 3 and error bars indicate SEM. No statistical significance in the difference between groups.

Next, we sought to investigate whether the *Smarca4*^mut^ and *Smarcc1*^mut^ lines had enhanced maturation capacity compared to scrambled controls. Transplantation of organoids into the colonic epithelium of adult mice has been used to define the differentiation potential of organoids derived from both the fetal and the adult intestine (*2*, *14*, *25*–*27*). To evaluate the maturation status of our mutated and control lines, we transplanted green fluorescent protein (GFP)–expressing experimental and control lines into severely immunocompromised animals that had been conditioned for transplantation by focal elimination of the endogenous epithelium (Fig. 4A). The engrafted material was isolated 3 weeks after transplantation and the GFP^+^-engrafted areas in all animals were assessed in a qualitative (Fig. 4B) and a quantitative manner (Fig. 4C). All three transplanted lines engrafted equally well (3/3 per line), and quantification of the engrafted areas revealed no significant differences in engraftment sizes across the three genotypes (Fig. 4C). In contrast to the in vitro analysis, we observed that engrafted patches from all three genotypes had similar numbers of CD117-expressing cells (Fig. 4, D and E). This suggested that the transplantation had affected the cellular identity and potentially the maturity of the engrafted material.

Previous transplantation experiments using organoids derived from the adult small intestine and colon have shown that the cellular identity of the engrafted material is restricted to the parental tissue origin of the transplanted cells (*25*–*27*). In contrast, engraftments of organoids derived from the fetal small intestine display features characteristic of the adult colon suggesting that these cells are not in the same manner regionally restricted (*14*). In line with previous observations (*14*), SCA1 expression is lost following the engraftment of both control and mutated cell lines (fig. S4). A feature discriminating the adult small intestine and colon is the organization of intestinal lymphatic vessels. In the adult small intestine, lymphatic LYVE1^+^ lacteals extend along the crypt-villus axis to the villus tip, whereas, in the adult colon, the lymphatic vessels are absent in the lamina propria between crypts and are instead organized as a plexus beneath the crypt base (*28*). Hence, the pattern of LYVE1^+^ lymphatic vessels can be used to probe the tissue identity of the engrafted material (*27*). Examination of the LYVE1^+^ patterns of lymphatic infiltration in GFP^+^-engrafted regions revealed that the transplanted *Smarca4*^mut^ and *Smarcc1*^mut^ lines had enhanced capacity to instruct their neighborhood and stromal tissues toward a small intestinal–like phenotype when compared to scrambled controls (Fig. 4, F and G).

To characterize the differentiation potential and the state of maturation of the engrafted cells, the engrafted material was stained for markers of differentiated cells with distinct expression patterns between the adult small intestine and colon. *Ulex Europaeus* agglutinin 1 (UEA-1) lectin recognizes sugar moieties on the surface of secretory cells with much higher cell numbers detected in the adult colon than in the adult small intestine (*29*). The engrafted scrambled control GFP^+^ areas contained more UEA-1^+^ cells when compared to regions of engrafted *Smarca4*^mut^ and *Smarcc1*^mut^ cells (Fig. 4, H and I). This demonstrates that the scrambled control cells acquired an identity that was similar to the recipient colonic epithelium upon engraftment. Markers for the adult small intestine, OLFM4, and colon, SATB2 (*30*), could not be detected in engrafted material across the three genotypes, suggesting that the engrafted cells had not undergone a complete maturation (fig. S4). We speculate that this is due to unresolved tissue damage, which is evident by the change in tissue architecture. Collectively, this suggests that SMARCA4 and SMARCC1 are important for safeguarding the fetal state and that SMARCA4 and SMARCC1 would normally impose an epigenetic barrier in fetal cells toward maturation.

### Smarca4/Smarcc1 maintain a Yap1-driven fetal-like state in intestinal progenitors

**Fig. 5.**
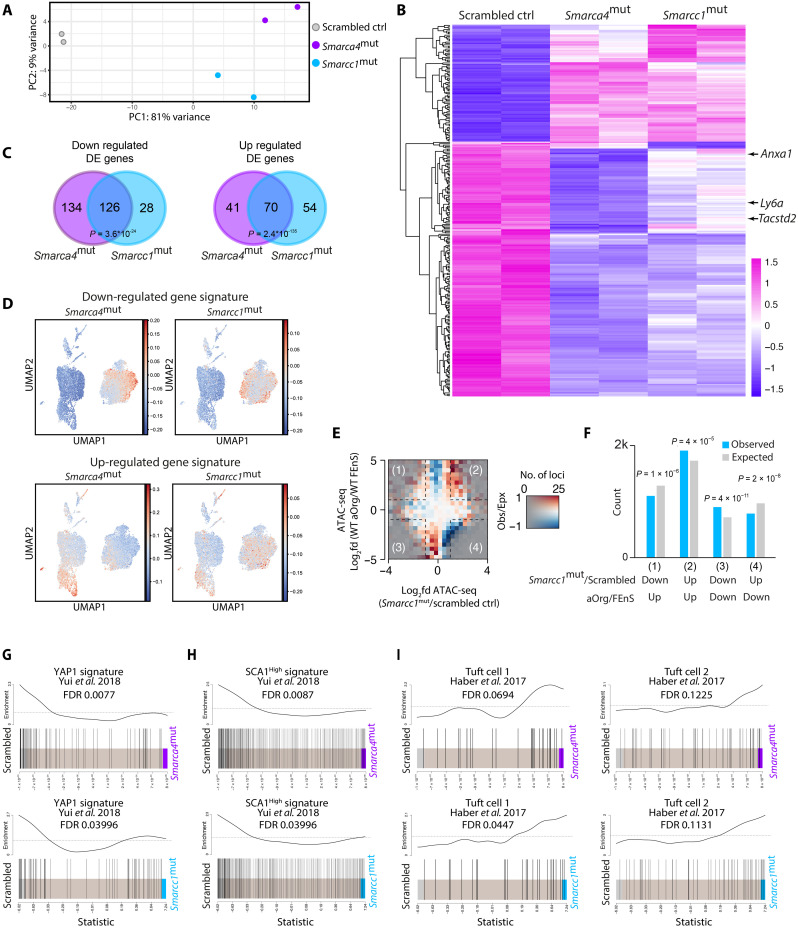
Mutations in *Smarca4* or *Smarcc1* induce similar maturation-associated transcriptional changes. (**A**) PCA plot of RNA-seq data from scrambled control, *Smarca4*^mut^, and *Smarcc1*^mut^ fetal organoids in biological duplicates. (**B**) Heatmap of DEGs between scrambled control, *Smarca4*^mut^, and *Smarcc1*^mut^ fetal organoids in biological duplicates (LFC >0.5; FDR <0.05). Expression of fetal-like markers *Ly6a* (SCA1), *Anxa1*, and *Tacstd2* (TROP2) is indicated by arrows. (**C**) Venn diagram of DEGs in *Smarca4*^mut^ and *Smarcc1*^mut^ fetal organoids compared to scrambled control (log_2_FC >0.5; FDR <0.05). Fisher’s exact test was used to calculate significance. (**D**) UMAP showing relative enrichment of genes down-regulated (top) and up-regulated (bottom) (LFC >0.5; FDR <0.05) in *Smarca4*^mut^ (left) and *Smarcc1*^mut^ (right) organoids compared to scrambled controls on scRNAseq data from wild-type (WT) FEnS and aOrgs (see Fig. 1B). (**E**) Matrix of the log_2_ fold differences in observed versus expected occurrences of loci in *Smarcc1*^mut^ organoids versus scrambled control fetal organoids (*x* axis) compared to assay for transposase-accessible chromatin sequencing (ATAC-seq) regions in WT control fetal and aOrgs (*y* axis) at enhancer regions (*n* = 57,353). Opacity is adjusted according to the number of loci with a given combination of ATAC-seq changes. The red color indicates more changes than expected, and the blue color indicates fewer changes than expected. (**F**) Bar plot of observed counts (blue) and expected counts (gray) of changed loci in the four squares of the matrix plot was shown in (E). *P* values from Bonferroni-corrected chi-square tests are indicated. (**G** and **H**) Gene set enrichment analysis (GSEA) of the YAP1-driven signature from the growth of aOrg in collagen versus Matrigel (G) or SCA1^High^ versus SCA1^Low^ cells from an in vivo model of experimental colitis (H) from Yui *et al*. (*17*), enriched in the transcriptome of the scrambled control line versus *Smarca4*^mut^ (top) or *Smarcc1*^mut^ (bottom). FDR value for negative enrichment in the mutant lines is indicated. (**I**) GSEA of two tuft cell gene sets from Haber *et al*. (*31*), enriched in the transcriptome of scrambled control lines versus *Smarca4*^mut^ (top) or *Smarcc1*^mut^ (bottom). FDR value for positive enrichment in the mutant lines is indicated.

To gain a deeper understanding of the molecular characteristics of the cell state imposed by disrupting *Smarca4* and *Smarcc1*, we performed bulk RNA-seq of the mutated lines along with the scrambled controls. The initial principal components analysis (PCA) revealed that the *Smarca4*^mut^ and *Smarcc1*^mut^ lines were distinct from the scrambled controls and clustered along principal component 1 (Fig. 5A). Thus, mutating *Smarca4* or *Smarcc1* enforces similar transcriptional changes. This was also evident upon analysis of DEGs between the three genotypes, where mutating *Smarca4* or *Smarcc1* led to the altered expression of many identical genes when compared to the scrambled controls (Fig. 5, B and C). This included the reduced expression of several markers of the fetal-like state beyond *Ly6a* (SCA1) (*14*, *15*) including *Anxa1* and *Tacstd2* (TROP2) (Fig. 5B). Moreover, when compiling signatures of genes up- or down-regulated in *Smarca4*^mut^ or *Smarcc1*^mut^ lines and integrating these signatures with the wild-type (WT) scRNA-seq dataset, it was evident that the genes down-regulated upon disruption of *Smarca4* or *Smarcc1* were predominantly expressed in FEnS (Fig. 5D). In contrast, genes up-regulated following *Smarca4* and *Smarcc1* disruption were expressed in adult cells of the secretory, enterocyte, and stem cell lineage (Fig. 5D). This lends further support to the function of SMARCA4 and SMARCC1 in maintaining an epigenetic barrier toward maturation in fetal immature cells.

As the canonical function of the SWI/SNF complex is nucleosomal positioning and controlling chromatin accessibility, we profiled the landscape of accessible chromatin in *Smarca4*^mut^, *Smarcc1*^mut^, and scrambled control lines by assay for transposase-accessible chromatin sequencing (ATAC-seq). ATAC-seq combined with cap analysis gene expression sequencing (CAGE-seq) using WT FenS and aOrgs has identified significant differences in chromatin accessibility and enhancer activity between the two cell states (fig.S4A) (*19*). This constituted a blueprint for assessing chromatin accessibility changes upon mutating *Smarca4* and *Smarcc1* when compared to the scrambled controls. When quantifying the genomic regions gaining accessibility in *Smarcc1*^mut^ cells compared to scrambled controls, we found a significant increase in regions that were enriched in aOrgs as compared to FEnS (Fig. 5, E and F). Similarly, regions with less ATAC signal in aOrgs compared to FEnS also displayed lower signal in *Smarcc1*^mut^ cells when compared to the scrambled controls (Fig. 5, E and F). This effect could also be observed in the *Smarca4*^mut^ lines, although less pronounced than in the *Smarcc1*^mut^ lines (fig. S4, B and C).

To gain insight into how *Smarca4* or *Smarcc1* facilitates an adult-like maturation, we performed gene set enrichment analysis (GSEA) using a number of transcriptional signatures associated with the fetal and adult states. We have previously demonstrated that the two transcriptional regulators downstream of mechano-sensing YAP1 and TAZ play key roles in reprogramming epithelial cells into a fetal-like state during regeneration (*17*) and that YAP1 is an essential gatekeeper of the immature state in FEnS (*19*). We detected significant enrichment of YAP1-driven signatures in scrambled control lines compared to *Smarca4*^mut^ and *Smarcc1*^mut^ lines (Fig. 5, G and H), demonstrating that mutating *Smarca4* and/or *Smarcc1* decreases the activity of YAP1-driven transcriptional programs. To uncover the nature of the proposed differentiated cell types emerging following decreased YAP1-activity, we performed GSEA using signatures associated with the distinct differentiated cell types in the adult small intestinal epithelium (Fig. 5I and fig. S5D) (*31*). Here, we detected significant enrichment of tuft cell–associated signatures in the *Smarcc1*^mut^ lines versus scrambled controls, and a similar tendency toward the enrichment of tuft cell–associated genes in the *Smarca4*^mut^ line (Fig. 5I).

Collectively, these results suggest that the *Smarca4*^mut^ and *Smarcc1*^mut^ lines exhibit transcriptional and epigenetic signatures distinct from their fetal-like origin, transitioning toward an adult-like state associated with decreased activity of YAP1-dependent transcriptional programs and enhanced propensity to generate tuft-like cells, further highlighting the role of SMARCA4 and SMARCC1 in safeguarding the fetal state.

### Enhanced tuft cell differentiation upon Smarca4 or Smarcc1 disruption

**Fig. 6.**
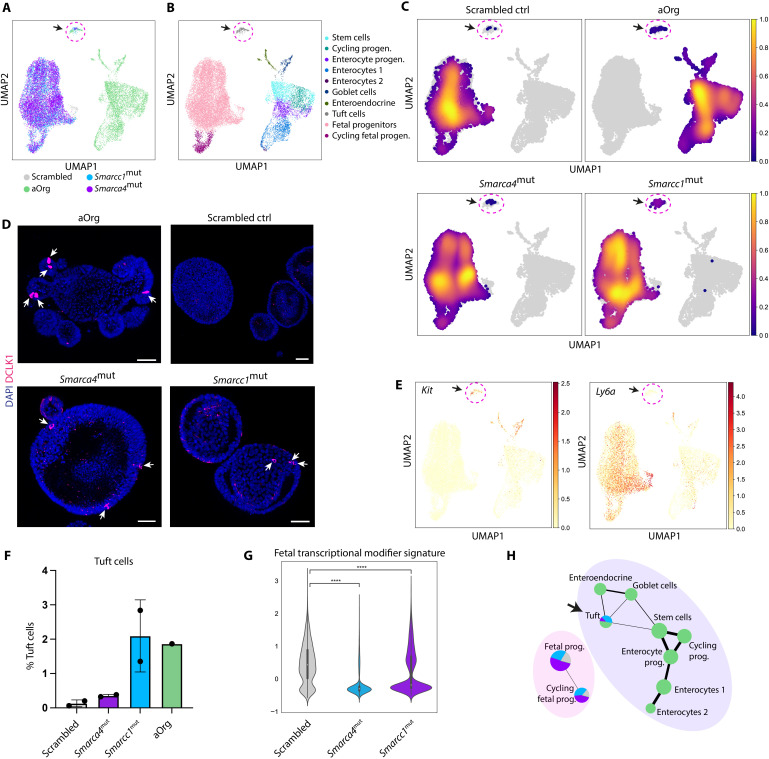
Increased frequency of adult tuft cells in *Smarca4*^mut^ and *Smarcc1*^mut^ organoids. (**A**) UMAP of scRNA-seq data from scrambled control (gray), *Smarca4*^mut^ (purple), and *Smarcc1*^mut^ (light blue) fetal organoids each in biological duplicates, as well as aOrg (green) from the WT dataset shown in Fig. 1. (**B**) UMAP showing cell type clusters. (**C**) UMAP showing the cell density of individual samples (scrambled control, aOrg, *Smarca4*^mut^, and *Smarcc1*^mut^) on the clusters in (B). The arrow and dotted circle indicate tuft cells [see (A) and (B)]. (**D**) Three-dimensional immunofluorescence images of DCLK1 expression in WT aOrgs, scrambled control, *Smarca4*^mut^, and *Smarcc1*^mut^ organoids. Arrows = DCLK1^+^ cells. Scale bars, 50 μm. (**E**) UMAP showing expression of *Kit* (left) and *Ly6a* (right). (**F**) Bar plot showing the percentage of tuft cells in scrambled control, *Smarca4*^mut^, and *Smarcc1*^mut^ fetal organoids in biological duplicates and WT aOrg. Error bars indicate SD. (**G**) Violin plot showing the expression of the fetal transcriptional modifier signature (Fig. 2B) in cells from the fetal progenitor cluster of scrambled control, *Smarca4*^mut^, and *Smarcc1*^mut^ organoids. ****P < 0.0001, Mann-Whitney *U* test using Bonferroni correction. (**H**) PAGA trajectory graph on clusters from (B). The color of the nodes represents the frequency distribution of samples (aOrg, scrambled control, *Smarca4*^mut^, or *Smarcc1*^mut^) in the different clusters. The size of the nodes is proportional to the number of cells in a cluster, and the thickness of the edges represents confidence in the presence of the transcriptional connection.

Although we detected transcriptional and epigenetic changes associated with a more mature state in the *Smarca4*^mut^ and *Smarcc1*^mut^ organoids compared to controls, the changes were not associated with a full conversion into budding organoids. To address the repertoire of adult-like cell types generated in vitro upon mutating *Smarca4* and *Smarcc1* at the single-cell level, we performed scRNA-seq of the scrambled controls, *Smarca4*^mut^, and *Smarcc1*^mut^ along with aOrgs. While most cells from the *Smarca4*^mut^ and *Smarcc1*^mut^ lines still clustered with the scrambled controls (Fig. 6A), we observed that a fraction of cells from the two mutant lines clustered together with adult tuft cells (Fig. 6, B and C, and fig. S6A), in line with our previous GSEA (Fig. 5H). These tuft cells could also be detected in our *Smarca4*^mut^ and *Smarcc1*^mut^ fetal organoids using three-dimensional (3D) immunofluorescence against the tuft cell marker DCLK1 (Fig. 6D). Similar to the identification of adult-like fetal cells in our scRNA-seq data from WT FEnS and aOrg (Fig. 1, B and C), the cells in the cluster expressing the tuft cell signature (fig. S6B) also expressed *Kit* but were negative for *Ly6a* expression (Fig. 6E). Looking at the proportion of fetal organoid-derived cells in the tuft cell cluster, most of these adult-like cells were derived from the *Smarca4*^mut^ and *Smarcc1*^mut^ lines (Fig. 6F). Moreover, we observed a significant reduction in the expression of the entire fetal transcriptional regulator signature (Fig. 6G) and YAP1-driven signatures (fig. S6C) in the *Smarca4*^mut^ and *Smarcc1*^mut^ lines compared to scrambled controls. Analyzing the transcriptional similarity furthermore revealed that the tuft cells derived from the mutant lines were transcriptionally similar to adult cells, suggesting that these cells have transitioned into an adult-like state (Fig. 6H).

Together, these results demonstrate that mutating *Smarca4* and/or *Smarcc1* enables in vitro cultures of fetal intestinal progenitors to lose their inherent immature characteristics, facilitating an increase in the fraction of cells differentiating into an adult-like state with a tuft cell identity.

## DISCUSSION

Here, we have successfully implemented a CRISPR-Cas9 screen in intestinal organoids targeting transcriptional and epigenetic regulators differentially expressed in epithelial cultures derived from distinct stages of tissue maturation. To our knowledge, this represents the first example of an in vitro CRISPR-Cas9 screen using immature fetal-like organoids to uncover regulators of tissue maturation, highlighting the versatility of organoid technology. Through single-cell analysis of fetal and aOrgs, we observed that a small number of fetal cells transition toward adult-like cells of the secretory lineage during steady-state conditions. We identified the genes *Smarca4* and *Smarcc1*, encoding components of the SWI/SNF complex, as barriers toward epithelial maturation, as disruption of these genes in vitro facilitated the loss of fetal-like markers and gain of secretory precursor and tuft cell markers, as well as the adoption of a transcriptional and epigenetic state akin to aOrgs. Our in vivo transplantation studies further suggested that the *Smarca4*^mut^ and *Smarcc1*^mut^ lines had a strengthened small intestinal identity when compared to the scrambled control line. As previous genetic studies have reported that *Smarca4* is involved in intestinal maturation, our screen successfully builds upon these observations and reports a yet unappreciated role for SMARCA4 in safeguarding cellular identity. The identification of SMARCC1 as a novel factor cooperating with SMARCA4 highlighted the involvement of the SWI/SNF complex in maintaining the intestinal progenitor state. This safeguarding putatively supports continuous epithelial growth during fetal development by skewing cell fate decisions toward self-renewing divisions.

While the reported genetic ablation of *Smarca4* during intestinal epithelial development in vivo led to a precocious generation of all differentiated secretory lineages (*23*), we only observed pronounced effects on a single population of adult tuft cells during in vitro disruption of *Smarca4* or *Smarcc1*. We speculate that the differences reflect the unique 3D culture conditions used for both fetal and aOrg cultures and that these conditions influence lineage differentiation. The cell state transition associated with disrupting *Smarca4* and *Smarcc1* might therefore not be sufficient to facilitate a full conversion toward the adult phenotype in vitro. This indicates that further barriers exist that prevent the maturation of the progenitor population and the generation or maintenance of the full repertoire of adult cell types in vitro. Alternatively, as most cells in our pooled *Smarca4*^mut^ and *Smarcc1*^mut^ cultures display minor insertions or deletions following CRISPR-Cas9–mediated disruption, the observed phenotype might be due to the disruption of specific interactions between SMARCA4 or SMARCC1 and other specific SWI/SNF complex members or transcription factors. Our findings, along with the reported in vivo phenotypes, suggest that the SWI/SNF complex plays a key role in the maturation of the intestinal epithelium during fetal development. Mechanistically, our findings suggest that a SWI/SNF complex containing SMARCA4 and/or SMARCC1 safeguards the immature fetal state by supporting a YAP1-driven transcriptional network essential for maintaining the fetal state (*19*). We further speculate that the SWI/SNF complex containing SMARCA4 and/or SMARCC1 facilitates ubiquitous activation of Notch (*23*), thereby inhibiting symmetry breakage necessary for the emergence of secretory cells (*16*). As the composition of the SWI/SNF complex changes during the progressive maturation of embryonic stem cells and during neuronal development (*22*), it is pertinent to speculate that similar dynamic changes in the composition of the SWI/SNF complex might govern the maturation of the intestinal epithelium by controlling the spatiotemporal accessibility of the chromatin landscape to repress or promote various gene regulatory networks allowing cell type specification. The epigenome of the secretory intestinal lineage has previously been demonstrated to be significantly distinct from intestinal stem cells (*32*), and hence, our findings suggest that the SWI/SNF complex might be involved in controlling some of these epigenetic state transitions as we also uncover an increased propensity to give rise to secretory CD117^+^ precursors and tuft cells upon *Smarca4* and *Smarcc1* disruption. In the current study, we limited our CRISPR-Cas9–based disruption to transcriptional regulators differentially expressed between FEnS and aOrgs. However, many transcription factors and epigenetic modifiers exert substantial changes to cellular identity following only modest changes in expression level or via posttranslational activity modulation. Future studies targeting a broader repertoire of factors in FEnS regardless of differential expression levels could help uncover additional regulators of tissue maturation. In addition, CRISPR-Cas9-based screens in aOrgs could help elucidate factors controlling adult intestinal cellular identity and maturation status.

We and others have recently demonstrated that the adult intestinal epithelium undergoes a process of transient reprogramming toward a more primitive epithelial cell state during tissue injury akin to the state stably adopted by FEnS (*17*, *33*, *34*). The expression of several constituents of the SWI/SNF complex, including *Smarcc1*, was recently demonstrated to be increased in patients suffering from Crohn’s disease when compared to the non-inflamed epithelium (*35*), suggesting that our findings on the role of SMARCA4 and SMARCC1 in controlling the maturation status of the intestinal epithelium might also extend to inflammatory conditions in the postnatal intestine. The SWI/SNF complex might thus represent a potential target for therapeutic intervention as alternating the epithelial maturation status could facilitate enhanced repair of lesions in the injured intestine. With the recent demonstration of fetal-like reprogramming during tissue injury (*17*, *33*) and in cancer (*36*, *37*), our findings might open novel therapeutic avenues for these pathologic conditions by modulating the maturation status of the intestinal epithelium.

## MATERIALS AND METHODS

### Animals

C57BL/6J mice (Taconic) were used for the derivation of adult and fetal organoids. NOD.Cg-Prkdc^scid^ Il2rg^tm1Sug^/JicTac (NOG) mice (Taconic) were used for transplantation assays. All animal procedures were approved by The Danish Animal Inspectorate.

### Fetal and adult organoid generation

aOrgs were generated as previously described (*12*). Briefly, the proximal small intestine of adult mice was isolated, luminal content was removed, and villi were scraped off. The remaining tissue were cut into small pieces using a scalpel and incubated in phosphate-buffered saline (PBS) with 2 mM EDTA on ice for 30 min. Crypts were isolated by vigorous shaking followed by filtering through a 70-μm filter mesh and subsequent centrifugation. Purified crypts were resuspended in Matrigel (Corning), which was allowed to solidify before the addition of advanced Dulbecco’s modified Eagle’s medium (DMEM)/F12, GlutaMAX supplemented with Hepes, penicillin/streptomycin (all from Thermo Fisher Scientific), epidermal growth factor (PeproTech; 50 ng/ml), noggin (PeproTech; 100 ng/ml), and conditioned R-spondin1 media (10%). Fetal organoids were generated as previously described (*14*). Briefly, the proximal small intestine from E16.5 fetuses was isolated and minced with a scalpel, followed by incubation in PBS supplemented with collagenase (Sigma-Aldrich; 125 μg/ml) for 45 min at 37°C, 900 rpm shaking. Isolated epithelial cells were seeded in Matrigel (Corning) and ENR media was added. Rho kinase inhibitor Y27632 (10 μM) was added during the initial days of culture. Cells were cultured under standard cell culture conditions at 37°C and 5% CO_2_. Experiments were performed on primary cell cultures below passage 25.

### Flow cytometry

Organoids were dissociated into single cells using mechanical disruption followed by incubation at 37°C for 15 min with TrypLE (Invitrogen) supplemented with deoxyribonuclease I (2.5 μg/ml) and Y-27632 (10 μM). After incubation, the cells were washed with advanced DMEM/F12 media with 10% adult bovine serum and pelleted by centrifugation. Single cells were incubated in 1% bovine serum albumin (BSA)/PBS before staining with SCA1-BV605 (1:200; D7; monoclonal Rat; BioLegend, catalog no. 108133; RRID: AB_2562275) and CD117-Pe-Cy7 (1:200; 2B8; monoclonal rat; BD Biosciences, catalog no. 558163; RRID: AB_647250) for 30 min on ice. Stained cells were washed three times with 0.1% BSA/PBS and incubated with 0.1% BSA/PBS and 4′,6-diamidino-2-phenylindole (DAPI) (0.2 μg/ml) before flow cytometric analysis or cell sorting.

### Single-cell RNA sequencing

WT fetal and aOrgs were isolated on day 5 after passaging as described under flow cytometry, and 20,000 live cells were sorted into 10 μl of ultraclean 0.8% BSA/PBS. scRNA libraries were prepared using 10x Genomics v3 Chemistry according to the manufacturer’s guidelines. Cells were loaded on the 10x Genomics single-cell chip for the formation of Gel Bead-in-Emulsions and followed by reverse transcriptase. cDNA was amplified with 10 polymerase chain reaction (PCR) cycles, while sample-indexing PCR was performed with 11 cycles. Libraries were diluted to 2 nM in the elution buffer (EB), and a final dilution of 1.7 pM was sequenced with the Illumina High Output 150 cycles kit on an Illumina NextSeq 500 sequencer.

For scRNA-seq of mutant FEnS (*Smarca4*^mut^, *Smarcc1*^mut^, and scrambled control), TotalSeq hashtag antibodies (A0301-A0305, BioLegend) were used to multiplex samples. Cells isolated from two individual organoid lines from each perturbation were sequenced. Cells were isolated on day 5 after passaging and dissociated into single cells. Each sample was incubated with 0.5 μg of unique hashtag antibody for 20 min on ice. Before sorting, cells were incubated with DAPI (0.2 μg/ml) for 10 min on ice. A total of 10,000 to 15,000 events per cell from each sample were sorted on a BD FACSAria III sorter (BD Biosciences) and pooled directly into four tubes. Single-cell libraries were prepared using 10x Genomics v3 Chemistry as described above with the implementation of hashtag libraries for demultiplexing. An additive primer (hashtag-oligo (HTO) primer) was added in the step for cDNA amplification. The hashtag cDNA and endogenous cDNA were separated after cDNA amplification using SPRIselect beads (Beckman Coulter) for size separation. Here, hashtag cDNA was isolated in the supernatant containing smaller DNA fragments and collected to generate the hashtag library following the protocol from Stoeckius *et al*. (*38*) and CITE-seq (https://cite-seq.com). Briefly, the supernatant was cleaned using SPRIselect beads (Beckman Coulter) and 30 ng of cDNA was used as input for PCR amplification (20 cycles). Illumina indexes for sequencing were subsequently added using Kapa HiFi HotStart Ready Mix (Roche Diagnostics). For the endogenous cDNA library, the 10x Genomics protocol was followed. The dual index kit set AA (10x Genomics) was used for sample indexing and 11 to 12 cycles were used during the final amplification step. The four hashtagged cDNA and four endogenous cDNA libraries were diluted to 2 nM and combined into two pooled libraries for sequencing (5% hashtag + 95% endogenous cDNA). The two final libraries for sequencing were further diluted to 1.7 pM before being sequenced with the Illumina High Output 150 cycles kit on an Illumina NextSeq500 sequencer.

### scRNA-seq data analysis

#### Processing of raw scRNA-seq reads

Raw reads were processed using Cell Ranger (v3.0.1) software from 10x Genomics (*39*) to generate bam files. Read alignment was based on the refdata-cellranger-GRCh38-3.0.0 reference downloaded from the 10x Genomics website (https://support.10xgenomics. com/). Raw base call files were demultiplexed into FASTQ files, and then aligned and filtered using cellranger’s mkfastq and count functions.

#### Quality control and filtering of cells

The bulk of the analysis was done in Python using Scanpy (*40*). The first steps were done on aOrg and fetal spheroid samples separately to filter out low-quality cells. The first metrics were per-cell distributions of spliced counts, unspliced counts, and genes. On the basis of visual tracing of a Gaussian curve, the cutoff values for those three characteristics were determined (specific to each sample). Second, cells with a high ratio of counts originating from mitochondrial features (more than 0.08%) were filtered out. Last, doublets (two cells erroneously sequenced together) were removed using Scrublet (*41*, *42*) and Doublet Detection packages. The expected doublet rate in Scrublet was set to 0.06, and DoubletFinder was used with default parameters. Cells predicted to be doublets by either of the tools were filtered out.

#### Data integration, normalization, and batch correction

After concatenating the samples together, genes expressed in less than five cells or with less than 20 counts were filtered out. Count data were normalized and log-transformed, without genes that account for more than 0.05 of total counts. Batch correction of the two samples was done with the mutual nearest neighbors algorithm implemented as the mnn_correct function in Scanpy’s API. We used the top 2000 highly variable genes; parameter k was set to 15 and var_adj to True.

#### Cell cycle annotation

Cell cycle stage annotation was done with the score_genes_cell_cycle function in the Scanpy package, based on published cell cycle signatures (*43*). It was done on the joint samples, but before the batch correction.

#### Regression, dimensionality reduction, and embedding

First, we used linear regression on the annotated S and G2/M scores to remove unwanted variation coming from the cell cycle. Next, the top 2000 highly variable genes were selected to be used for the rest of the analysis. For further dimensionality reduction, we first used PCA and then Uniform Manifold Approximation and Projection. Cell neighborhood was calculated with 30 principal components.

#### Clustering

Initial unsupervised Leiden clustering with a high-resolution parameter gave us multiple clusters, which were then combined judging by the expression of canonical cell type markers (*Olfm4*: Stem cells; *Lrmp*: Tuft cells; *Scgn*: enteroendocrine cells; *Ca1*: enterocytes; *Muc2*: goblet cells; *Ly6a*: fetal cells) and the presence of cells in the cell cycle (positive S or G2/M score, as calculated before). This iterative process of subclustering and cluster merging gave rise in the end to the informative clusters that are presented in the figures.

#### Differential expression

Wilcoxon rank sum test was used to detect DEGs using scanpy’s rank_genes_groups function. Cells in each cluster (cell type) were compared to the remaining cells in the samples. For the plot of cluster markers (fig. S1A), only genes expressed in at least 25% of the cells of a given cluster are shown, and in no more than 75% of cells outside of that cluster. The genes also have to have at least 1.5-fold higher expression between the analyzed cluster and the rest of the cells.

#### Trajectory analysis

Trajectories were inferred using Partition-based graph abstraction (PAGA) from Scanpy (*44*) assessed on cell type clusters by approximating each cluster to a single node and connecting them with edges based on similarity.

#### Gene signature enrichment

Gene set enrichment signatures were calculated with the score_genes function in Scanpy. The score plotted is the average expression of the set of genes subtracted with the average expression of a reference set of genes randomly selected from all the genes in the sample. It indicated the enrichment (or depletion) of given genes expression in a cell.

#### Hashtag demultiplexing

Samples of mutant FEnS (*Smarca4*^mut^, *Smarcc1*^mut^, and scrambled controls) have been separated (demultiplexed) using the HTODemux function from the R package Seurat (v3). Before demultiplexing, hashtag counts have been filtered, normalized using the function NormalizeData, and scaled using the function ScaleData. Cells with more than 20,000 HTO counts have been filtered out. NormalizeData has been run with parameters normalization.method set to CLR and margin set to 2. HTODemux and ScaleData have been run with default parameters. Cells annotated as doublet or negative have been removed from the dataset and singlet cells have been divided into the final samples based on the assigned hashtag. Around 10% of cells were removed that way.

### sgRNA library generation

Ten to 12 sgRNAs were designed per gene to target functional domains in the experimental 226 up-regulated transcriptional regulators in FEnS and in the 50 positive control genes (*20*, *21*). One thousand scrambled control sgRNAs were selected from the mouse GeCKO library (*45*). The sgRNA oligos were manufactured by Custom Array Inc. (a member of GenScript), and the library was cloned as a pool following an established protocol (*46*). In short, the sgRNA vector (Addgene, #52963) was digested with the restriction enzyme BsmBI, and sgRNA oligos were amplified through 10 PCR cycles. The oligos and vector were assembled using NEBuilder HiFi DNA Assembly Kit (New England BioLabs) with a molar ratio of 1:10 of vector versus insert. The cloned library was transformed into Stellar Competent bacteria (Takara) and purified using Maxi-Prep (Qiagen). To verify the complexity of the sgRNA library, sgRNAs were amplified through PCR and sequenced using the Illumina platform. The sgRNA plasmid library was transfected into human embryonic kidney (HEK) 293T cells for lentiviral production. MOI was calculated by transducing HEK293T cells with two dilutions of the stock vial, and the number of infected cells was assessed through DAPI staining after puromycin selection.

### Adherent cultures

Established fetal organoids were dissociated into small clumps of cells with mechanical disruption and seeded in wells coated with human recombinant laminin 511 (ln511) (BioLamina) in ENR media supplemented with nicotinamide (Nic) (10 mM) (ENR-Nic) and Y-27632 (10 μM). Cells could be cultured long-term as adherent cultures with media (ENR-Nic) change every 2 to 3 days. Cells were passaged every 5 to 6 days and dissociated following incubation with Accutase (Merch Millipore) at 37°C for 5 to 10 min. Dissociated cells were washed with advanced DMEM/F12 and seeded as single cells in ENR-Nic media which were supplemented with Y-27632.

### Organoid transduction

Single cells from WT fetal organoids were seeded as adherent cultures on ln511-coated wells in ENR-Nic media supplemented with Y-27632. After 4 to 8 hours, lentivirus-containing ENR-Nic media supplemented with Y-27632 and Polybrene (1:1000) was added to the cells for a 12-hour incubation, and the medium was subsequently changed to ENR-Nic. Selection of successfully transduced cells was initiated after 1.5 to 2 days by the addition of either puromycin (3 μg/ml) or blasticidin (5 μg/ml) to the medium.

### CRISPR-Cas9 screen

WT fetal organoids were transduced with Cas9-blasticidin plasmid (Addgene, #52962) as described under organoid transduction and expanded as adherent cultures. Cells were treated with blasticidin (5 μg/ml) in normal organoid media for two to three passages. A total of 20 × 10^6^ Cas9-expressing fetal cells were transduced with the sgRNA puromycin library as described under organoid transduction at a MOI of 0.3. Cells were isolated 40 hours after transduction for time point T = 0 and, at this point, the puromycin (3 μg/ml) selection was initiated. The next day, transduced cells were dissociated with Accutase and >16.5 × 10^6^ cells were seeded in Matrigel at a seeding density of 40,000 cells in 60 μl of Matrigel droplets. With the library size of 4106 sgRNAs and a previously estimated organoid forming efficiency of 10 to 15%, the fold coverage of individual sgRNAs at the time of Matrigel seeding was >400×. The medium was supplemented with nicotinamide, Y-27632, and Chir99021 (3 μM) for the first 3 days along with puromycin and blasticidin. Time point T = 1 was isolated at day 10 after Matrigel seeding. At each passage, ^1^/_3_ of the cells were passaged at a ratio of 1:3 for T = 2 and later for T = 3, while ^2^/_3_ of the culture underwent cell sorting as described under flow cytometry. Cells were stained for SCA1 and CD117 and three cell populations were sorted: CD117^Pos^, CD117^Neg^SCA1^Low^, and CD117^Neg^SCA1^High^. Sorted cells were pelleted by centrifugation and the resulting pellets were frozen at −80°C.

DNA from all time points was isolated using DNeasy Blood and Tissue Kit (Qiagen), and inserted sgRNAs were amplified from genomic DNA using KAPA HiFi HotStart Library Amplification Kit (Kapa Biosystems) with 20 cycles of PCR (see the oligo list in table S1). A second PCR reaction was used to add sample unique Illumina sequencing barcodes. The amplified sgRNA libraries were subjected to electrophoresis using agarose gels and the amplified band was isolated using the Gel Extraction Kit (Qiagen) and cleaned with the DNA Clean & Concentrator kit (Zymo Research). The concentrations of the sgRNA libraries were measured using a KAPA Library Quantification kit (Kapa Biosystems) and diluted to 2 nM before libraries were pooled. A final dilution of 1.7 pM pooled libraries was sequenced with the Illumina High Output 75 cycles kit and sequenced on the Illumina NextSeq500 sequencer.

### Analysis of the CRISPR-Cas9 screen

The screen sequencing data were analyzed with MAGeCK (model-based analysis of genome-wide CRISPR-Cas9 knockout) (*47*). Briefly, the sequencing reads were mapped to the sgRNA design library, and read counts were calculated for all individual sgRNAs. Seven guides with an extreme number of mapped reads were excluded from further analysis. Screen hits were identified by integrating FCs and *P* values of individual sgRNAs targeting the same gene using robust ranking aggregation for comparison between any two conditions to identify depleted or enriched sgRNAs between time points and cell populations. The counts were normalized on the basis of 100 nontargeting sgRNAs. All sgRNAs were present at T = 0 and positive control sgRNAs targeting essential genes were rapidly depleted. Significant screen hits were selected on the basis of FDR <0.05 and absolute LFC >1.

### Generation of sgRNA targeted FEnS

Single-gene–targeted FEnS were generated in duplicates. WT FEnS were transduced with the same Cas9-blasticidin construct as used in the screen using adherent cultures as described under organoid transduction. sgRNAs were cloned into a sgRNA-eGFP-puro plasmid (modified construct of Addgene #57827 with puromycin resistance inserted instead of Cas9) using BsmBI sites, and lentivirus carrying the sgRNA constructs was produced in HEK293T cells. Cas9-blasticidin–expressing FEnS were transduced with the sgRNA-eGFP-puro plasmid as described under organoid transduction, and selection with puromycin was started after 2 days. sgRNA-expressing cells were dissociated and seeded in 3D Matrigel 1 day later and ENR-Nic-Y27632 media supplemented with Chir99021, blasticidin, and puromycin. Cells were sorted on the BD FACSAria III sorter for Sca1 expression 9 days after Matrigel embedding. *Smarca4* and *Smarcc1* targeted cells were sorted for Sca1^Low^ and Sca1^High^ expression. Scrambled sgRNA-targeted cells were used as control cells. Sorted cells were seeded in Matrigel and ENR-Nic media supplemented with Chir99021 and Y-27632. DNA was isolated with the DNeasy Blood and Tissue Kit (Qiagen) from the transduced cells two passages after the initial sorting. The targeted genomic regions were PCR-amplified (see the oligo list in table S1) and Sanger sequenced. Synthego’s Inference of CRISPR Edits tool was used to analyze the in-dels generated as well as the knockout score.

### RNA sequencing

RNA was isolated from duplicate lines of scrambled controls, *Smarca4*^mut^, and *Smarcc1*^mut^ organoids using the RNeasy Micro Kit (Qiagen). RNA-seq libraries were generated with the TruSeq RNA Library Prep Kit (Illumina). The concentration and quality of the library were measured using Qubit and Bioanalyzer. The library was diluted to 2 nM and a final concentration of 1.7 pM was sequenced with the Illumina High Output 75 cycles kit on an Illumina NextSeq 500 sequencer.

### RNA-seq analysis

Base-calling and demultiplexing were performed using BaseSpace, and the 76–base pair (bp) single-end raw reads were quality-assessed with FastQC (v0.11.7), Fastq Screen (v0.11.4), and trimmed using Trimmomatic v0.32 (*48*). The trimmed reads were aligned to the mouse genome (mm10 assembly excluding noncanonical chromomes) using STAR (*49*) (v2.7.3a) in a two-pass mode guided by a RefSeq gene annotation. Bam file indices were created using SAMTools (*50*) (v1.10). The mapped reads were assigned to genes with featureCounts (*51*) generating a count table. After alignment, the package RSeQC (*52*) was used to calculate and check various QC measures such as genebody coverage and TIN score (analog to RIN score). In R (v3.5.1), the DESeq2 (v1.22.1) (*53*) (R Team 2014, www.r-project.org/) package was used for statistical analysis of the count data comparing the different groups. For the PCA plot, the calculations used the 500 genes with the highest variation across samples as input. The R package pheatmap (v1.0.10) was used to generate a heatmap of *Z* scores of DEGs.

### Gene set enrichment analysis

GSEA was performed with the edgeR fry function (*54*). Gene signatures from Yui *et al*. (*17*) included a Yap1-driven response of aOrg to growth in collagen versus Matrigel, and a signature of SCA-1^High^ cells derived following in vivo injury of the adult mouse colon versus the homeostatic tissue. From Haber *et al.* (*31*), we used signatures of differentiated cell types in the adult mouse small intestinal epithelium. The results are visualized with a barcode plot where genes are ranked from left to right by increasing log fold change comparing scrambled controls to *Smarcc1*^mut^ or *Smarca4*^mut^. FDR-corrected *P* values were used for statistics.

### ATAC-sequencing

Duplicate lines of scrambled controls, *Smarca4*^mut^, and *Smarcc1*^mut^ were isolated and dissociated to single cells as described under flow cytometry, and 50,000 live cells were sorted in 200 μl of 0.1% BSA/PBS for the generation of ATAC-seq libraries (*55*). Briefly, sorted cells were lysed and incubated with Tn5 transposase (Illumina) for 30 min at 37°C for DNA tagmentation. The tagged DNA was PCR-amplified with sequence-compatible and barcoded primers for a total of 13 cycles. The concentration and quality of the libraries were analyzed with Qubit and Bioanalyzer. The libraries were diluted to 2 nM in EB buffer and a final dilution of 1.5 pM was sequenced with the Illumina High Output 150 cycles kit on an Illumina NextSeq 500 sequencer.

### ATAC-seq analysis and visualization

blc2fastq (v2.20.0.422) was used for base-calling and demultiplexing. The raw 76-bp paired-end (PE) reads were first trimmed for Nextera transposase adapter sequences using Trimmomatic in palindrome mode. FastQC (v0.11.7) of reads before and after trimming confirmed the removal of any 3′-end adapter sequences while also clearly showing the known insertion Tn5 motif in the 5′-end. FastQ Screen (v0.11.4) was furthermore used to check for sample contamination. The trimmed PE reads were mapped to the mouse genomes (mm10 assembly, canonical chromosomes only) using bowtie2 v.2.2.9 with default settings except -k 2 -X 2000 --no-mixed --no- discordant and lastly converted to bam format using SAMTools (v1.10). After sorting (SortSam) and labeling duplicates (MarkDuplicates) with Picard tools and adding an NH tag (number of reported alignments), reads were filtered to exclude unmapped, multimapping, and mitochondrial reads (SAMTools view -f 2 -F 4 and custom filter). The filtered bam files were converted to bed format using bedtools bamtobed.

Mapped reads were imported from bed files as ‘Datasets’ into EaSeq (*56*) (v. 1.2, http://easeq.net) for subsequent analysis using its integrated tools and default settings where nothing else is stated. A list of previously identified organoid enhancers including values from differential accessibility analysis as previously described (*19*) was imported as a ‘Regionset.’ ATAC-seq signal was quantified in 1-kbp windows centered at the previously identified regions using the ‘Quantify’ tool with all normalization disabled to give raw read counts and the Regionset was exported. Based on this, a subset of regions was filtered having a signal above the 0.9 fractile in at least two of eight samples (two replicates of Smarca4, two replicates of Smarcc1, and four replicates of negative controls) and termed ‘filtered enhancers.’ Differentially accessible regions were identified using DeSeq2 (*53*) (R Team 2014) at the raw read counts in the filtered enhancers. DeSeq2 output was exported as a text file, merged with coordinates and other previously generated data, and reimported in EaSeq as a ‘Regionset.’ Volcano plots were visualized on the basis of the DeSeq2 output using the ‘Scatter’ plot type with the axis set to show linear values. The matrix of observed versus expected changes in ATAC-seq signal was generated by first making a 2D histogram of LFCs of the previously analyzed aOrgs versus fetal organoids (*19*) and the LFCs from the differential accessibility analysis in *Smarcc1* and *Smarca4* mutant organoids. The settings for this plot were altered to show both axes on a linear scale, reduce the number of bins on both axes to 24, and disable intrapolation. The plot was selected and the observed versus expected matrix was generated and visualized using the ‘Obs/Exp’ tool.

### Transplantation

Fetal organoids were isolated from 8 to 10 wells with gentle pipetting and Matrigel was removed using Recovery Solution (Corning) by incubating for 10 to 15 min on ice. Isolated organoids were resuspended in 200 μl of PBS with 5% Matrigel. Transplantation into mice was performed using a previously published protocol (*27*). Briefly, NOG mice (13 weeks, female, Taconic) were anesthetized with 2% isoflurane before the procedure. The luminal content of the colon was flushed with PBS before an electric pulsating device, soaked in prewarmed 0.5 M EDTA, was used to brush crypts off on one side of the colon. The organoids resuspended in 200 μl of PBS were gently disrupted and infused into the conditioned colon. Glue (Histo-acryl, B. Braun) was added to the anal verge and left for 3 hours to avoid the ejection of the organoid suspension and thereby enhance the engraftment of the infused material. The engrafted tissue was analyzed 3 weeks after transplantation using a Leica M165FC microscope with a Leica DF310 FX camera to acquire macroscopic images.

### Immunofluorescent staining of frozen sections

Tissue was embedded in optimal cutting temperature compound (CellPath) and frozen at −80°C and sections of 7 μm were cut on a Leica Microsystem cryostat. Sections were washed with PBS three times before blocking in 1% BSA/PBS supplemented with adult bovine serum (10%) and Triton X-100 (0.2%) (blocking buffer) for 1 hour at room temperature. Subsequently, primary antibodies were incubated in a blocking buffer overnight at 4°C. The following primary antibodies were used: anti-GFP (1:300 dilution; polyclonal rabbit antibody; Invitrogen, catalog no. A-11122; RRID: AB_221569), anti-KIT(1:200 dilution; polyclonal goat antibody; R&D Systems, catalog no. AF1356; RRID: AB_354750), anti-LYVE1 (1:300 dilution; polyclonal goat antibody; R&D Systems, catalog no. AF2125; RRID: AB_2297188), UEA-1 (1:1000 dilution; DyLight649; Vector Laboratories, catalog no. DL-1068-1), anti-SCA1 (1:200 dilution; monoclonal rat antibody; R&D Systems, catalog no. BAM1226; RRID: AB_2070040), anti-OLFM4 (1:200 dilution; monoclonal rabbit antibody; Cell Signaling Technology, catalog no. 39141; RRID: AB_2650511), anti-SATB2 (1:100 dilution; monoclonal rabbit antibody; Abcam, catalog no. ab92446; RRID: AB_10563678), anti–beta-catenin [1:500 dilution; monoclonal mouse immunoglobulin G1 (IgG1) antibody; BD Biosciences, catalog no. 610154; RRID: AB_397555]. Sections were, following overnight incubation, washed three times for 5 min in PBS before incubation for 1 to 2 hours with secondary antibodies and DAPI (0.2 μg/ml) diluted in blocking buffer at room temperature protected from light. The following secondary antibodies were used (all 1:500): Alexa Fluor (AF) 488 polyclonal donkey anti-rabbit antibody (Invitrogen, catalog no. A-21206; RRID: AB_2535792), AF555 polyclonal donkey anti-goat antibody (Invitrogen, catalog no. A-21432; RRID: AB_2535853), AF647 polyclonal donkey anti-rabbit (Invitrogen, catalog no. A-31573; RRID: AB_2536183), AF555 polyclonal goat anti-mouse IgG1 antibody (Invitrogen, catalog no. A-21127, RRID: AB_2535769), AF555 polyclonal goat anti-rat (Invitrogen, catalog no. A-31572, RRID: AB_162543). Immunofluorescence images were acquired using a confocal microscope (Leica TSC SP8 or Zeiss LSM880) and analyzed in ImageJ.

### Image quantification

Image quantification was performed in ImageJ (Fiji). For the quantification of GFP^+^ engraftment areas, image files containing the GFP^+^ channel were smoothened and next subjected to thresholding to obtain a binary mask of the GFP^+^ signal. The area of the resulting GFP^+^ mask was measured using the “analyze particles” function. CD117^+^ and UEA1^+^ cells were counted manually, and the final cell count number was normalized to the GFP^+^ area. The area of infiltrating LYVE1^+^ lymphatic vessels in engrafted regions was performed as previously described (*27*). Briefly, the GFP^+^ engraftment area was outlined by a region of interest (ROI), the outside of the ROI was cleared, and the area of the LYVE1^+^ channel was quantified as described for the GFP^+^ signal above.

### 3D staining of FEnS and aOrg

Organoids were grown in Matrigel in a 96-well imaging plate (Sigma-Aldrich). The plate was centrifuged at 800*g* for 10 min at 4C before fixation with 4% paraformaldehyde for 1 hour on ice. Organoids were incubated with a blocking buffer for 1 hour at room temperature, before incubation overnight at 4C with primary antibodies suspended in a blocking buffer. The following primary antibodies were used: anti-SCA1 (1:400 dilution; monoclonal rat antibody; R&D Systems, catalog no. BAM1226; RRID: AB_2070040), anti-KIT (1:200 dilution; monoclonal rabbit antibody; Cell Signaling Technology, catalog no. 3074; RRID: AB_1147633), anti–beta-catenin (1:500 dilution; monoclonal mouse IgG1 antibody; BD Biosciences, catalog no. 610154; RRID: AB_397555), and anti-DCLK1 (1:400 dilution; polyclonal rabbit antibody; Abcam, catalog no. ab31704; RRID: AB_873537). Each well was washed three times for 5 min with PBS, before incubation for 1 to 2 hours with secondary antibodies and DAPI (0.2 μg/ml) diluted in a blocking buffer at room temperature and protected from light. The following secondary antibodies were used (all 1:500): AF555 polyclonal goat anti-rat (Invitrogen, catalog no. A-31572, RRID: AB_162543), AF488 polyclonal goat anti-mouseIgG1 (Invitrogen, catalog no. A-21121, RRID: AB_2535764), AF647 polyclonal donkey anti-rabbit antibody (Invitrogen, catalog no. A-31573, RRID: AB_2536183), AF555 polyclonal goat anti-mouse IgG1 antibody (Invitrogen, catalog no. A-21127, RRID: AB_2535769), and AF555 polyclonal donkey anti-rabbit (Invitrogen, catalog no. A-31572, RRID: AB_162543). Wells were washed three times with PBS and incubated with RapiClear (SunJin Lab) for 1 to 2 hours before imaging on a confocal microscope (Leica TSC SP8).

## Notes

View/request a protocol for this paper from *Bio-protocol*.
