# Supplementary material for: An organoid-based CRISPR-Cas9 screen for regulators of intestinal epithelial maturation and cell fate
Source: Sci Adv. 2023 Jul 12;9(28):eadg4055. doi: 10.1126/sciadv.adg4055 (PMC10337909; doi:10.1126/sciadv.adg4055)
Supplement: Supplementary file 1 — Figs. S1 to S6 Table S1 [file sciadv.adg4055_sm.pdf]

Science Advances
AAAS

Supplementary Materials for

An organoid-based CRISPR-Cas9 screen for regulators of intestinal epithelial maturation and cell fate

Stine L. Hansen *et al.*

Corresponding author: Kim B. Jensen, kim.jensen@sund.ku.dk

Sci. Adv. **9**, eadg4055 (2023)
DOI: 10.1126/sciadv.adg4055

This PDF file includes:

Figs. S1 to S6
Table S1

Supplementary Text

Figure S1

A

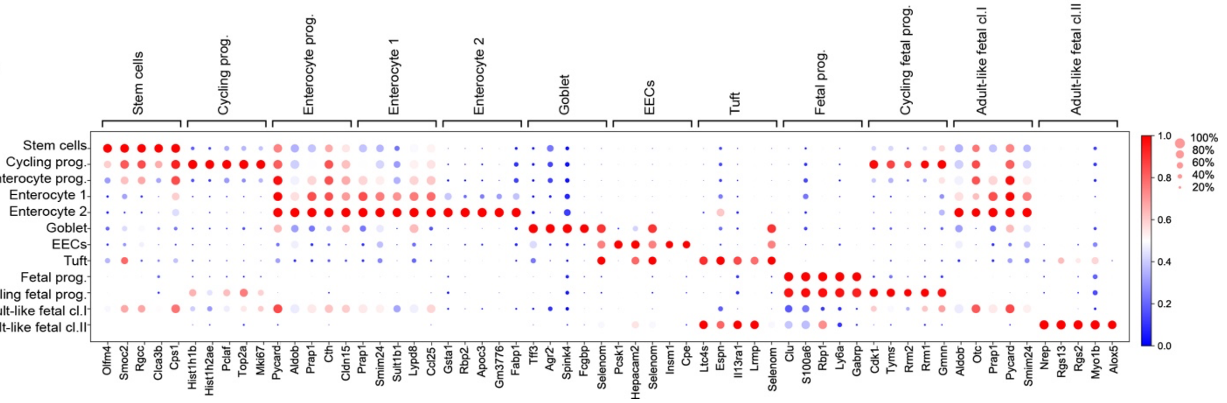

Figure S1:

(A) Heatmap of gene expression across cell clusters shown in Fig.1B.

Figure S2

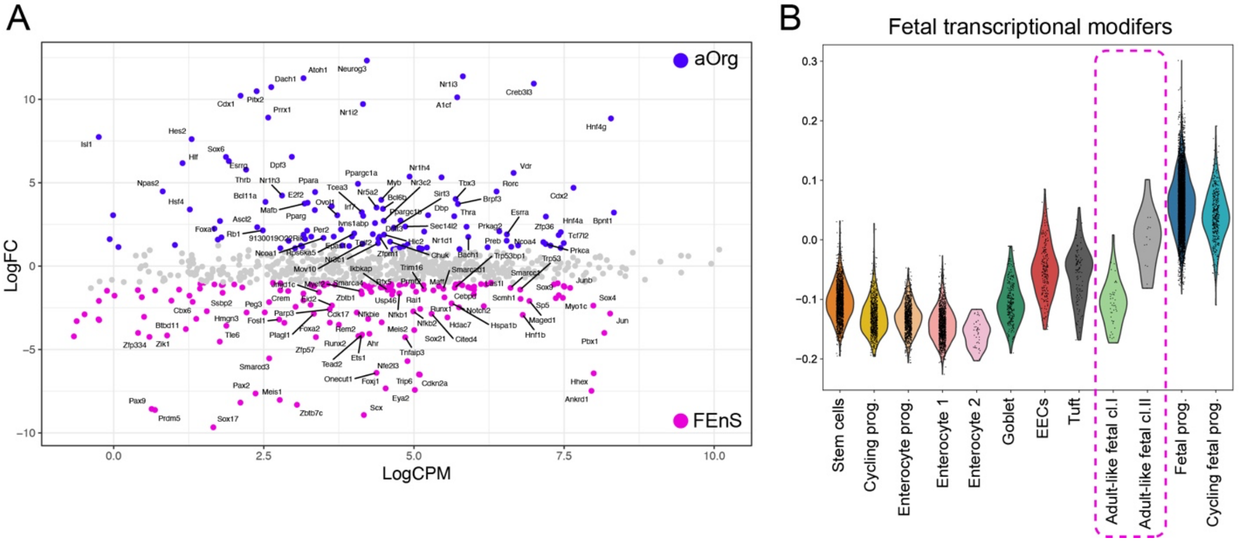

Figure S2:

(A) MA plot showing DE genes between FEnS (pink) and aOrg (blue). x-axis: average logCPM (counts per million), y-axis: logFC **(B)** Violin plot showing expression of the fetal transcriptional signature in scRNA-seq cell clusters.

Figure S3

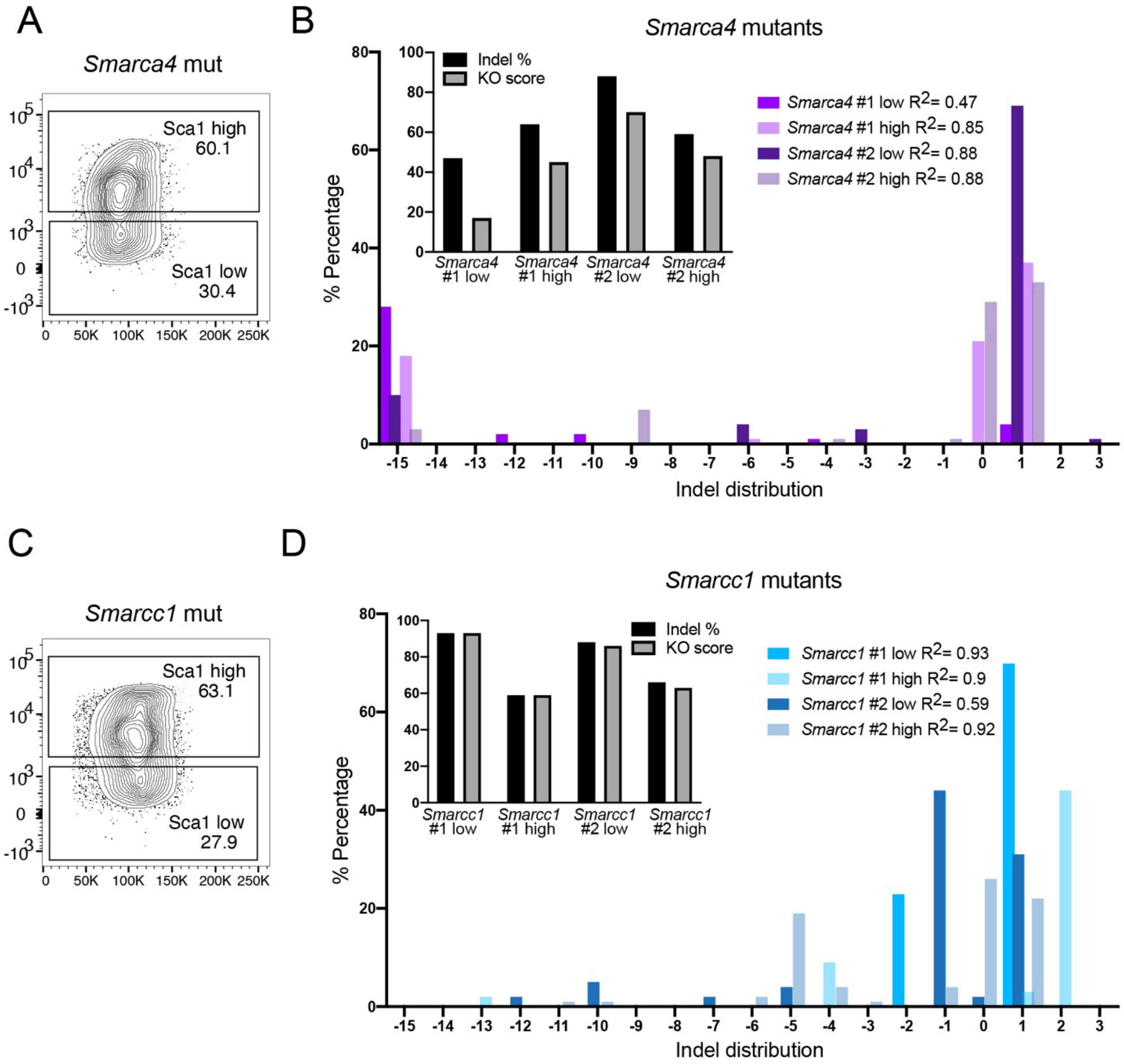

Figure S3:

(A) Representative FACS plot of Sca1 expression in one out of two *Smarca4* mutant organoid lines one passage after lentiviral sgRNA transduction. $Sca1^{High}$ and $Sca1^{Low}$ cells were sorted and cultured separately. **(B)** Indel analysis based on Sanger sequencing of duplicates of *Smarca4* mutant organoids, $Sca1^{High}$ and $Sca1^{Low}$ sorted, generated with Synthego´s ICE tool. Indel distribution from -15 to +3 base pair position around expected cut site from specified cell populations is shown. R^2 indicates how well the predicted indel distribution and KO score align with the provided sequence for analysis. Inserted plots show indel percentage and KO score of

indicated cells. **(C)** Representative FACS plot of Sca1 expression in one out of two *Smarcc1* mutant organoids one passage after lentiviral sgRNA transduction. $Sca1^{High}$ and $Sca1^{Low}$ cells were sorted and cultured separately. **(D)** Indel analysis based on Sanger sequencing of duplicates of *Smarcc1* mutant organoids, $Sca1^{High}$ and $Sca1^{Low}$ sorted, generated with Synthego´s ICE tool. Indel distribution from -15 to +3 base pair position around expected cut site from specified cell populations is shown. R^2 indicates how well the predicted indel distribution and KO score align with the provided sequence for analysis. Inserted plots show indel percentage and KO score of indicated cells

Figure S4

A

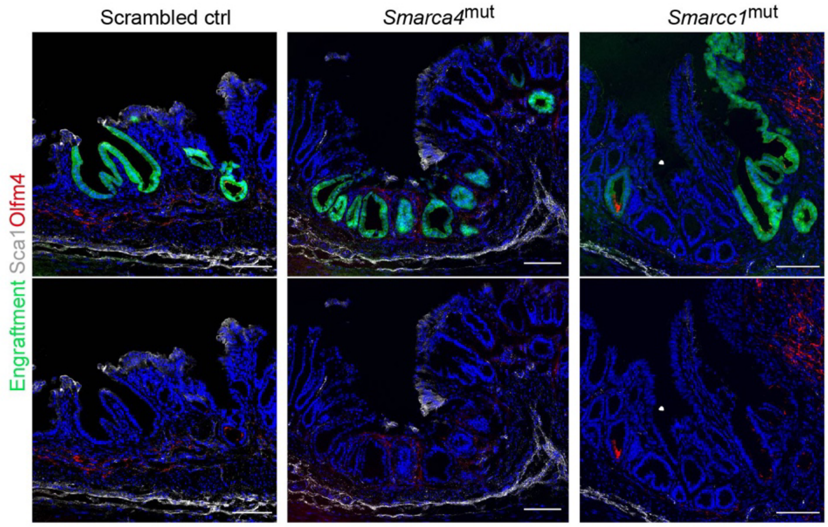

B

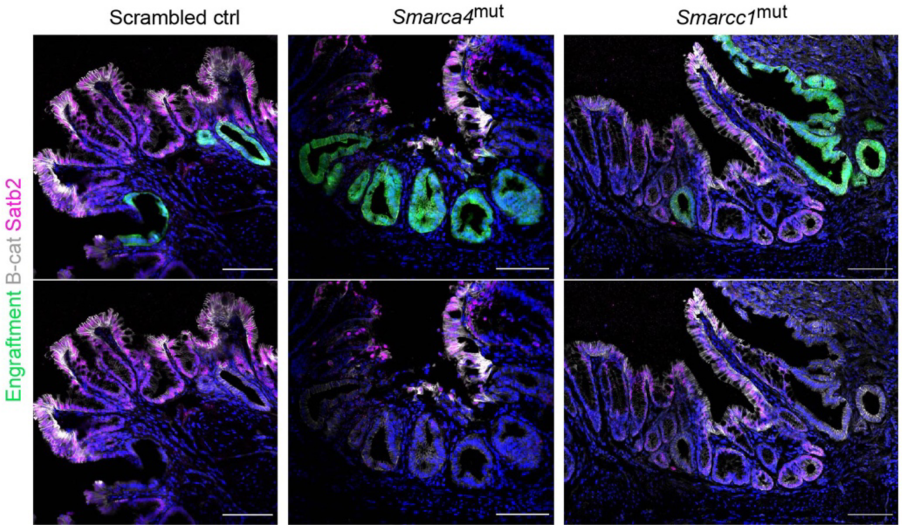

Figure S4:

(A) Immunofluorescence staining of Olfm4 (red) and DAPI (blue) in engrafted GFP^{+} (green) tissue of scrambled control, *Smarca4*^{mut} and *Smarcc1*^{mut}. Scale bar 100 µm. **(B)** Immunofluorescence staining of Satb2 (pink), β-catenin (white) and DAPI (blue) in engrafted GFP^{+} (green) tissue of scrambled control, *Smarca4*^{mut} and *Smarcc1*^{mut}. Scale bar 100 µm.

Figure S5

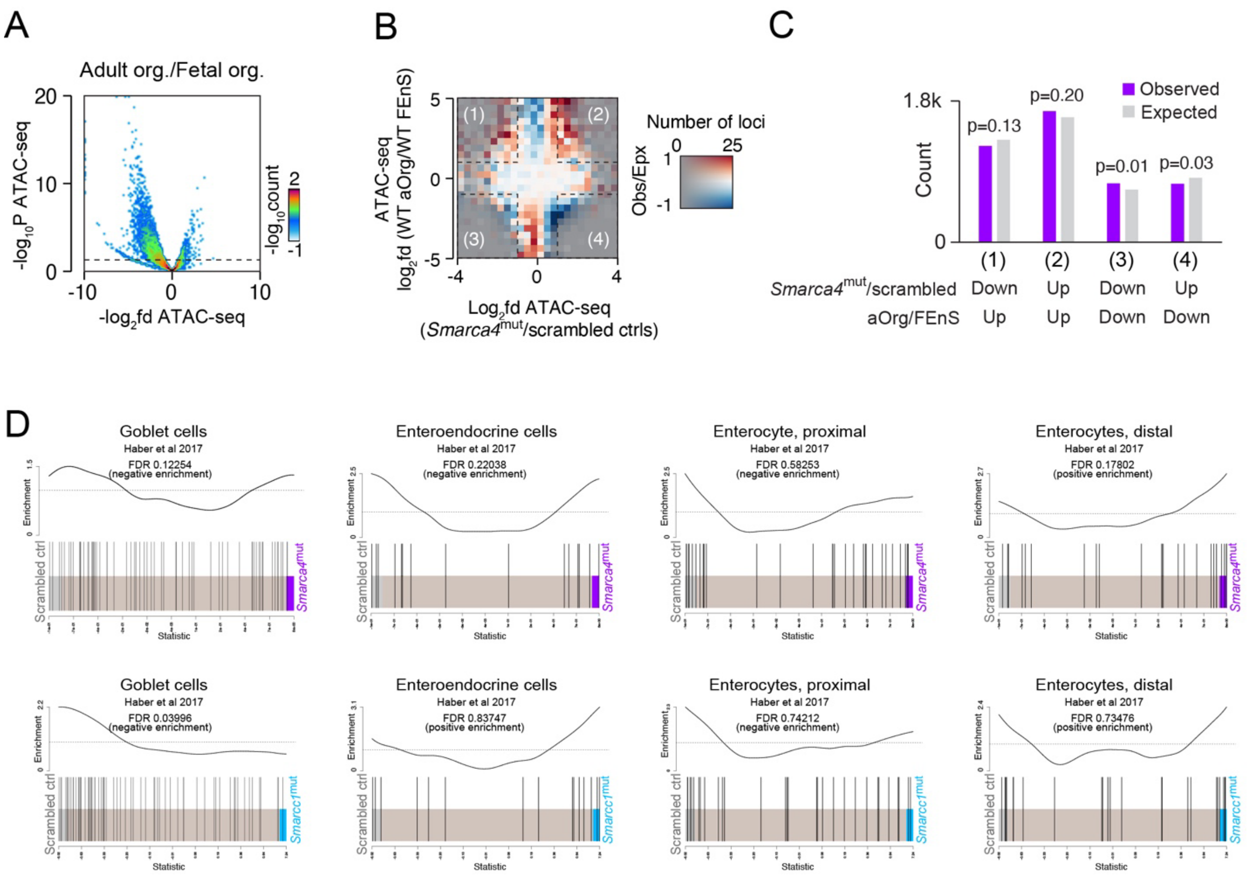

Figure S5:

(A) Volcano plot of differential ATAC-seq regions of adult organoids vs FEnS at FEnS-specific enhancer regions (n=7,492). P-values were Benjamini-Hochberg corrected for multiple testing, and the dashes line indicates the 0.05 significance threshold. **(B)** Matrix of the log2 fold difference in observed vs expected occurrences of loci in *Smarca4*^{mut} organoids vs scrambled control FEnS (x-axis) compared to ATAC-seq regions in WT control FEnS and adult organoids (y-axis) at enhancer regions (n=57,353). Opacity is adjusted according to the number of loci with a given combination of changes. Red colour indicates more changes than expected, and blue colour indicates less changes than expected. **(C)** Bar plot of observed counts (purple) and expected counts (grey) of changed loci in the four squares of the matrix plot shown in (B). P-values from Bonferroni-corrected chi-square tests are indicated. **(D)** GSEA of differentiated cell types (Goblet cells, enteroendocrine cells, enterocytes from proximal and distal small intestine) enriched in the transcriptome of scrambled control line and *Smarca4*^{mut} (top) or *Smarcc1*^{mut} (bottom). FDR value for positive or negative enrichment in the mutant lines are indicated.

Figure S6

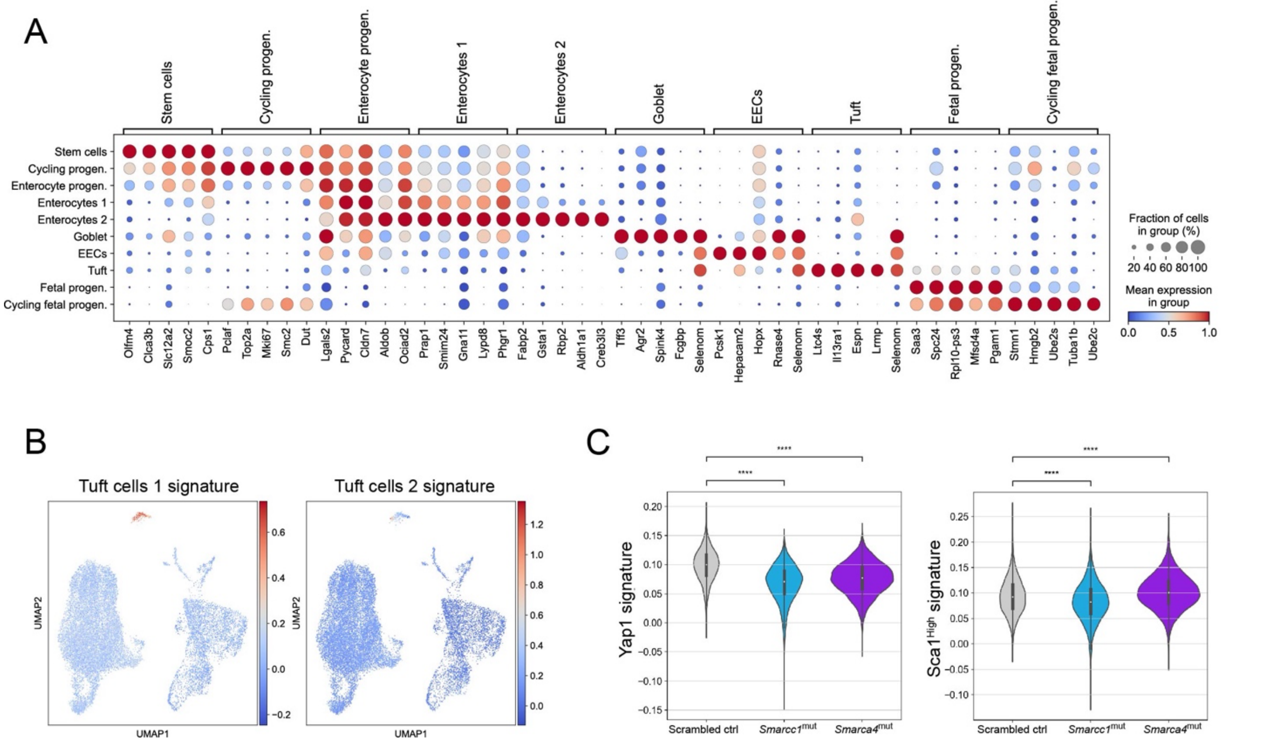

Figure S6:

(A) Heatmap of gene expression across cell clusters shown in Fig. 6B. **(B)** UMAP showing tuft cell 1 (left plot) and tuft cell 2 (right plot) gene signatures. **(C)** Violin plot showing expression of Yap1-driven gene signature (left plot) and Sca1^{High} gene signature (right plot) from Yui et al. 2018 in cells from scrambled control, *Smarcc1*^{mut} and *Smarca4*^{mut} organoids. ****=p<0.0001, Mann-Whitney U test Bonferroni correction.

	Name	Sequence	
Oligo PCR	sgRNA-oligo_PCR_Fw	GGCTTTATATATCTTGTGGAAAGGACGAAACACCG	
	sgRNA-oligo_PCR_Rev	CTAGCCTTATTTTAACTTGCTATTTCTAGCTCTAAAAC	
sgRNA library seq	sgRNA-seq_PCR1_Fw	TCTTGTGGAAAGGACGAAACACCG	
	sgRNA-seq_PCR1_Rev	TCTACTATTCTTTCCCCTGCACTGT	
	sgRNA-seq_PCR2_Fw	AATGATACGGCGACCACCGAGATCTACACTCTTTCCCTACACGAC GCTCTTCCGATCTTTAGGCTCTTGTGGAAAGGACGAAACACC	
	sgRNA-seq_PCR2_Rev	CAAGCAGAAGACGGCATACGAGAT**N***GTGACTGGAGTTCAGAC GTGTGCTCTTCCGATCTATTCTTTCCCCTGCACTGTACC	N* 6 basepair index
sgRNA	Smarca4 sgRNA	TGGTTCTCGCCACCGCAAGG	
	Smarcc1 sgRNA	GCTTGCCGGCGAAACCTGAC	
Indel analysis	Smarca4_seq_fw1	GCTGGGTCAGTCCCCAAAAT	
	Smarca4_seq_rev2	CCTCCAGGGGAATTTGCTGAT	
	Smarcc1_seq_fw1	TGCTTAGCAACCAAGACCACA	
	Smarcc1_seq_rev1	ATTCCTTCCCCCAGGAGTCA	

Table S1: Oligo list

Sequence of oligos used.
